# Lowered Insulin Signalling Ameliorates Age-Related Sleep Fragmentation in *Drosophila*

**DOI:** 10.1371/journal.pbio.1001824

**Published:** 2014-04-01

**Authors:** Athanasios Metaxakis, Luke S. Tain, Sebastian Grönke, Oliver Hendrich, Yvonne Hinze, Ulrike Birras, Linda Partridge

**Affiliations:** 1 Max Planck Institute for Biology of Ageing, Cologne, Germany; 2 Institute of Healthy Ageing, and Department of Genetics, Evolution and Environment, University College London, London, United Kingdom; Stanford University, United States of America

## Abstract

Sleep fragmentation, particularly reduced and interrupted night sleep, impairs the quality of life of older people. Strikingly similar declines in sleep quality are seen during ageing in laboratory animals, including the fruit fly *Drosophila*. We investigated whether reduced activity of the nutrient- and stress-sensing insulin/insulin-like growth factor (IIS)/TOR signalling network, which ameliorates ageing in diverse organisms, could rescue the sleep fragmentation of ageing *Drosophila*. Lowered IIS/TOR network activity improved sleep quality, with increased night sleep and day activity and reduced sleep fragmentation. Reduced TOR activity, even when started for the first time late in life, improved sleep quality. The effects of reduced IIS/TOR network activity on day and night phenotypes were mediated through distinct mechanisms: Day activity was induced by adipokinetic hormone, dFOXO, and enhanced octopaminergic signalling. In contrast, night sleep duration and consolidation were dependent on reduced S6K and dopaminergic signalling. Our findings highlight the importance of different IIS/TOR components as potential therapeutic targets for pharmacological treatment of age-related sleep fragmentation in humans.

## Abstract 2

Reduced insulin signaling improves sleep quality in flies and is protective against age-related sleep deterioration.

## Author Summary

Sleep is essential for human health, but the quality of this fundamental physiological process declines with age and reduces quality of life. We therefore investigated the mechanisms by which ageing impairs sleep. We used the fruit fly *Drosophila*, whose sleep has many features in common with that of humans, including the age-related decline in quality. We examined the role of the insulin/IGF (IIS) and TOR signaling network, which has an evolutionarily conserved role in ageing. We found that flies with reduced IIS activity had improved sleep quality at night and higher activity levels by day. Importantly, day activity and night sleep were regulated through distinct mechanisms—day activity by the key IIS transcription factor dFOXO, adipokinetic hormone, and octopaminergic signalling—whereas night sleep was mediated through TOR and dopaminergic signalling. Surprisingly, acute inhibition of TOR, by rapamycin, even in old flies, improved sleep quality, suggesting that age-related sleep decline is reversible even after it has commenced. Given the high evolutionarily conservation of IIS and TOR function, our results implicate potential therapeutic targets to improve sleep quality in humans.

## Introduction

Increased human life expectancy has brought with it an increased burden of age-related loss of function and pathology, including sleep disorders, which affect ∼50% of the population over 65 years old. Older people often have difficulty in initiating and maintaining sleep, associated with reduced quality of life, poor health, and increased mortality [1]. Sleep in *Drosophila* and humans has marked similarities, making the fruit fly a powerful genetic model in which to study sleep syndromes [2]. Several drugs and signalling pathways affect sleep in flies and mammals in a similar manner, including arousal-promoting dopaminergic signalling [3],[4]. Like humans, flies are more active by day and sleep mostly during the night, and this pattern deteriorates with age: duration of day sleep increases and of night sleep decreases, and sleep bouts become shorter and more often interrupted by waking periods, known collectively as sleep fragmentation [5].

We hypothesised that evolutionarily conserved mechanisms that ameliorate ageing itself could also ameliorate the deterioration in sleep quality in older individuals. Mutants that reduce insulin/insulin-like growth factor (IGF) signalling (IIS) can extend healthy lifespan in the nematode worm *Caenorhabditis elegans*, the fruit fly *Drosophila*, and the mouse, and may also ameliorate human ageing [6]–[8]. In *C. elegans* and *Drosophila*, extension of lifespan by reduced IIS requires the single forkhead Box O (FOXO) transcription factor [9]–[12]. Effects of IIS on ageing are also mediated through its interaction with the Target of Rapamycin (TOR) signalling pathway. The TOR pathway interacts with IIS in part through phosphorylation of the AKT kinase [13] and TOR also regulates translation, through 4E-BP and S6K activity [14], and autophagy, through *atg* genes [15]. Down-regulation of TOR signalling by the TOR-specific inhibitor rapamycin extends lifespan in flies and mammals [16],[17]. It is not clear if reduced IIS and/or TOR activity can delay neural and behavioural senescence, because increased activity in the nervous system itself can be neuroprotective in specific disease states [18], and extended lifespan is not invariably accompanied by amelioration of age-related loss of behavioural function [19]. However, IIS regulates processes involved in CNS function and brain ageing, such as oxidative stress response, autophagy, and protein homeostasis [20], suggesting that its manipulation could improve neural function and hence behaviour during ageing.

We characterised the sleep and activity of two long-lived *Drosophila* strains with down-regulated IIS, *dilp2-3,5* mutants, which lack three genes encoding *Drosophil*a insulin-like peptides [21], and flies expressing a dominant-negative form of the insulin receptor [22]. We found that reduced IIS affected both day and night activity and sleep, through distinct mechanisms. Reduced IIS induced day activity, through dFOXO and adipokinetic hormone (AKH), and octopaminergic signalling, while it increased night sleep duration and consolidation through TOR, S6K, and dopaminergic signalling. IIS mutants were resistant to the age-related sleep deterioration seen in the controls. Moreover, acute pharmacological inhibition of TOR with rapamycin, even late in life, was sufficient to reverse age-related fragmentation of night sleep.

The conserved role of IIS and TOR signalling in multicellular organisms makes its components promising pharmacological targets for treatment of human sleep disorders and syndromes.

## Results

### Reduced IIS Affected Activity and Sleep Patterns But Not Circadian Rhythm

**Figure 1:**
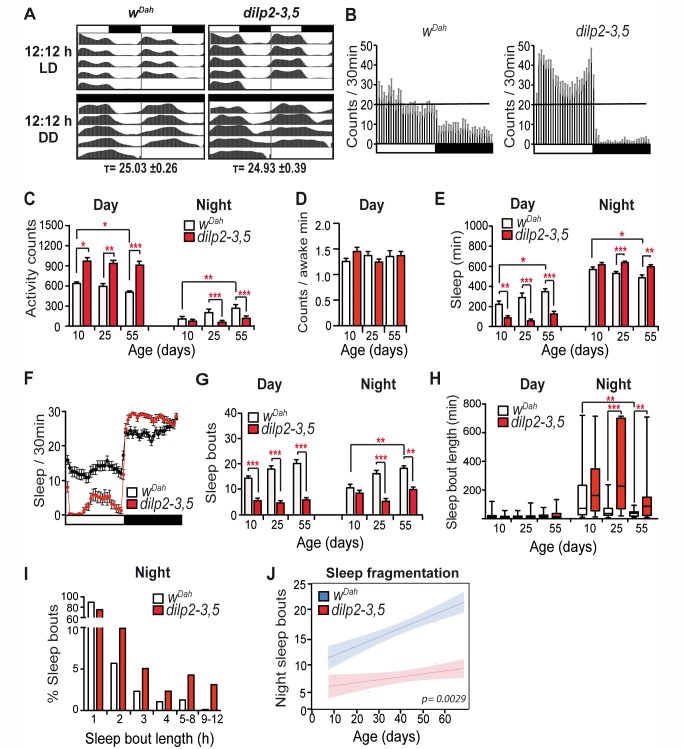
Reduced IIS affected activity and sleep and ameliorated age-related sleep fragmentation. (A) Locomotor activity over 9 d of *w^Dah^* control and *dilp2-3,5* mutant flies under 12∶12 h LD and constant darkness 12∶12 h DD (*n = *12, age 20 d). Mean free running period (τ) in DD ± s.e.m. (B) Average activity count data (30 min bins) under 12∶12 h LD conditions (25 d *w^Dah^ n = *48, *dilp2-3,5 n = *31). (C) *dilp2-3,5* mutants were more active during the day and less active during the night compared to controls. (D) There was no significant difference in wakefulness (average activity per waking minute). (E) *dilp2-3,5* mutants slept more at night and less during the day than controls. (F) Minutes of sleep per 30 min (25 d *w^Dah^ n = *48, *dilp2-3,5 n = *31). (G) Day and night sleep of *dilp2-3,5* mutants were interrupted by fewer waking periods compared to controls. (H) *dilp2-3,5* flies had longer sleep bouts during the night. (I) Longer sleep bouts were more prevalent in *dilp2-3,5* mutants (age 25 d). (J) *w^Dah^* control flies, but not *dilp2-3,5* mutants, show a significant age-related increase in night sleep bouts (age 10 d, 25 d, 45 d, 55 d, and 65 d). (B–F) *w^Dah^* , *n* = 31, 43, 46, 26, and 31 for ages 10 d, 25 d, 45 d, 55 d, and 65 d, respectively; *dilp2-3,5*, *n* = 31, 31, 32, 29, and 43 for ages 10 d, 25 d, 45 d, 55 d, and 65 d, respectively. Kruskal Wallis test with Dunn's multiple comparison (selected pairs). ***p<0.001, **p<0.01, and *p<0.05. Error bars represent s.e.m.

To investigate the effects of reduced IIS on activity and sleep, we measured activity patterns of long-lived, *dilp2-3,5* mutant flies and controls [21]. Activity and circadian rhythm can be correlated [23], and we therefore first measured both of them under 12∶12 h light∶dark (LD) and constant dark (DD) conditions over a 5-d period. Control, *w^Dah^* flies showed typical circadian rhythmicity, which was unaltered in *dilp2-3,5* mutants (Figure 1A). However, in the mutants day activity was significantly increased, whereas night activity was significantly reduced, a pattern that was maintained as the flies aged (Figure 1B–C). Although day activity was higher in the *dilp2-3,5* mutants, wakefulness (average activity per awake minute [24]) was not significantly altered (Figure 1D), suggesting that *dilp2-3,5* mutants had a greater number of active periods during the day.

Sleep in flies is defined as a resting period of no activity that lasts for 5 min or longer. While asleep, flies have a characteristic posture and increased arousal threshold, and longer sleep bouts include a deep sleep state characterised by electrophysiological changes and regulated by molecules involved in synaptic plasticity and pruning [23],[25],[26]. At all ages tested, *dilp2-3,5* mutants slept more at night and less by day than did controls (Figure 1E–F). In addition, they had fewer waking periods, and hence sleep bouts, during both day and night, and longer night sleep bouts (Figure 1G–H). Longer sleep periods occurred mainly in the *dilp2-3,5* mutants (Figure 1I). Thus, reduced IIS induced more day activity periods but increased both night sleep duration and sleep consolidation, and these phenotypes were already evident in young flies.

To determine if the unaltered circadian rhythmicity and the activity and sleep phenotypes of *dilp2-3,5* mutants are a general feature of reduced IIS in *Drosophila*, we measured these traits in flies in which IIS was down-regulated by ubiquitous expression (driven by *da-Gal4* driver) of a dominant-negative form of the single fly IIS receptor (*da-Gal4/UAS-INR^DN^*) [22]; all of the phenotypes seen in the *dilp2-3,5* mutant were present (Figure S1A–G).

Consistent with previous studies [5], sleep fragmentation increased with age in *w^Dah^* control flies. Day sleep increased while night sleep declined (Figure 1E), and the number of day and night sleep bouts increased and night sleep bout duration decreased with age (Figure 1G–H). In contrast, sleep fragmentation showed little or no increase with age in *dilp2-3,5* mutants. Night sleep duration did not change (Figure 1E), day sleep duration did not change either (Figure 1E), while day and night sleep bouts did not increase with age (Figure 1G). Generalized linear modelling (GLM) indicated that all aspects of sleep fragmentation increased significantly less with age in the *dilp2-3,5* mutants than in controls: total day and night sleep, *p* = 0.0019 and *p*<0.0001, respectively, and day and night sleep bouts, *p* = 0.0017 and *p* = 0.0029, respectively. Reduced IIS thus rescued the age-related sleep fragmentation of old flies (Figure 1J).

Age-related night sleep fragmentation was also ameliorated in *da-Gal4/UAS-INR^DN^* flies. GLM indicated that, while day behaviours did not differ, age-related night sleep fragmentation of *da-Gal4/UAS-INR^DN^* flies increased less with age than in both genetic controls: total night sleep, *p*<0.0001 and *p* = 0.0003 *da-Gal4/+/UAS-INR^DN^/+*, respectively; night sleep bouts *p = *0.011 and *p* = 0.016 *da-Gal4/+/UAS-INR^DN^/+*, respectively (Figure S1G). Amelioration of age-related sleep fragmentation is thus a general feature of reduced IIS in *Drosophila*.

### Increased Day Activity of *dilp2-3,5* Mutants Was Dependent on Light

In diurnal species, such as humans and *Drosophila*, LD cycle entrains circadian rhythms, which regulate the timing of sleep and arousal, with light promoting arousal and dark promoting sleep [27]. To determine the role of IIS in these responses, we measured activity and sleep behaviours of *dilp2-3,5* mutants and controls in response to DD conditions (Figure S2). The *dilp2-3,5* mutants and controls showed similar responses in night behaviours, with increased activity, and reduced sleep and sleep bouts under DD conditions. Interestingly, the response in day behaviours differed significantly between *dilp2-3,5* mutants and controls. Control flies showed no change in day activity, a slight decrease in sleep duration, and a decrease in sleep bouts. In contrast, day activity of *dilp2-3,5* mutants was reduced to the point where it did not differ from that of controls, sleep and sleep bout length increased, while sleep bout number was unaltered under DD conditions (Figure S2). A normal LD cycle was therefore required for the daytime, but not the nighttime, activity and sleep phenotypes of *dilp2-3,5* mutants.

### Loss of FOXO Affected Day Behaviours of *INR^DN^* Flies

**Figure 2:**
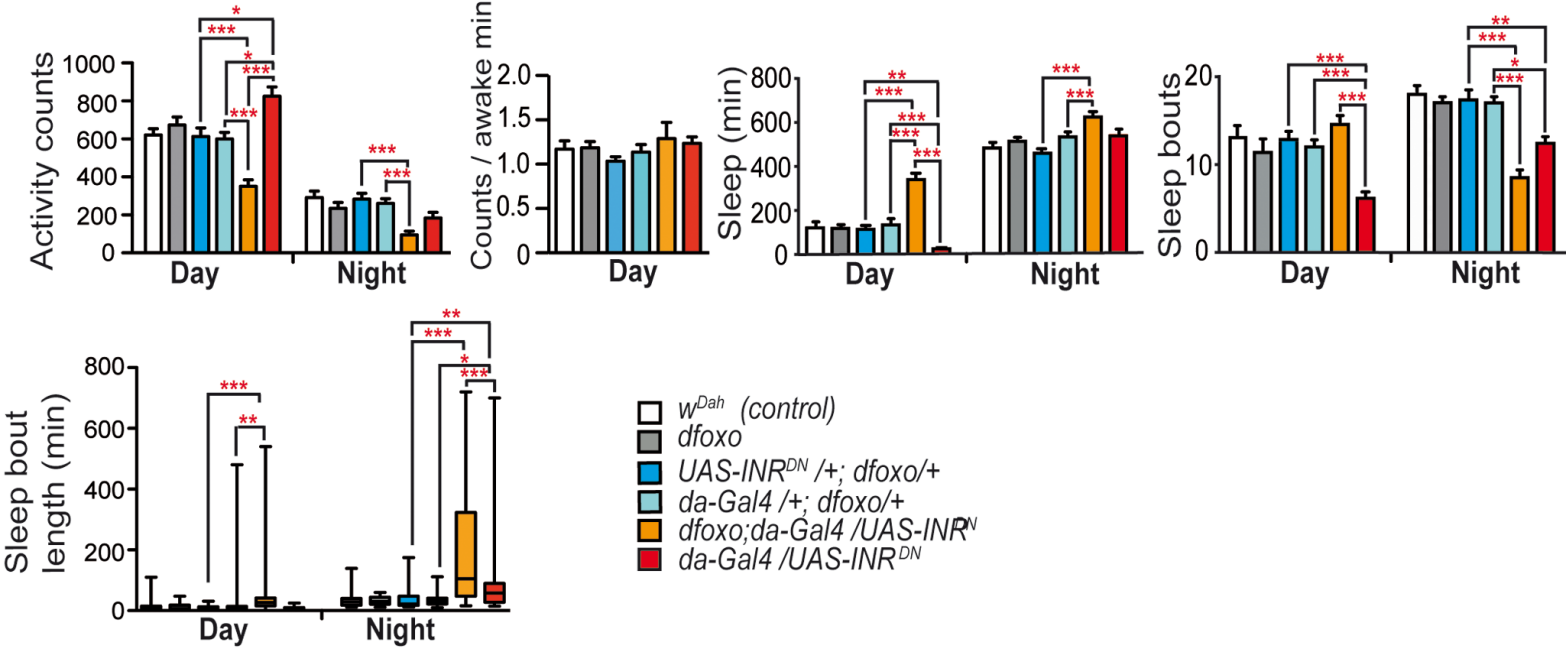
*dfoxo* affected daytime activity and sleep phenotypes of *INR^DN^* flies. Loss of *dfoxo* in *da-Gal4/UAS-INR^DN^* flies but not in wild-type flies (age 20 d) decreased day activity but had no effect on night activity, had no significant effect on wakefulness (average activity per waking minute), increased day sleep duration but had no effect on night sleep duration, and reverted the low sleep bout phenotype of *da-Gal4/UAS-INR^DN^* flies by day but not at night and increased night sleep bout duration. *dfoxo* indicates the *dfoxo^Δ94^*allele (*n = *35 for all genotypes). Kruskal Wallis test with Dunn's multiple comparison test (selected pairs). ***p<0.001, **p<0.01, and *p<0.05. Error bars represent s.e.m. Independent experiments verifying activity and sleep phenotypes of *INR^DN^;dfoxo* double mutants are shown in Figure S3.

To identify molecular mechanisms that mediated the activity and sleep phenotypes of IIS mutants, we investigated the role of the transcription factor dFOXO, which is essential for lifespan extension by reduced IIS in *Drosophila* [12]. *dfoxo/dilp2-3,5* double null mutants are lethal, but *dfoxo/INR^DN^* double mutants are viable. In a wild-type background, loss of *dfoxo* function did not induce any activity or sleep phenotypes (Figure 2 and Figure S3). However, loss of *dfoxo* in *INR^DN^* flies specifically affected day, but not night, phenotypes (Figure 2 and Figure S3). *INR^DN^;dfoxo* double mutants showed strongly reduced day activity and increased day sleep duration, without changes in wakefulness, and the number of day sleep bouts did not differ significantly between *INR^DN^;dfoxo* double mutants and controls (Figure 2 and Figure S3). Reduced IIS thus affects day sleep and activity through dFOXO, but night sleep through a different route.

### Reduced IIS Induced Day Hyperactivity through AKH and Octopaminergic Signalling

Like glucagon in mammals, AKH is a peptide that acts antagonistically to insulin to increase lipolysis and glycogenolysis in the fly fat body [28], the equivalent of mammalian liver and white adipose tissue. AKH expression is increased in larvae and adults lacking brain insulin-producing cells [29], and AKH has been shown to increase activity in flies [30]. We therefore hypothesised that reduced IIS could act through increased AKH release and *AkhR* function to regulate day activity.

**Figure 3:**
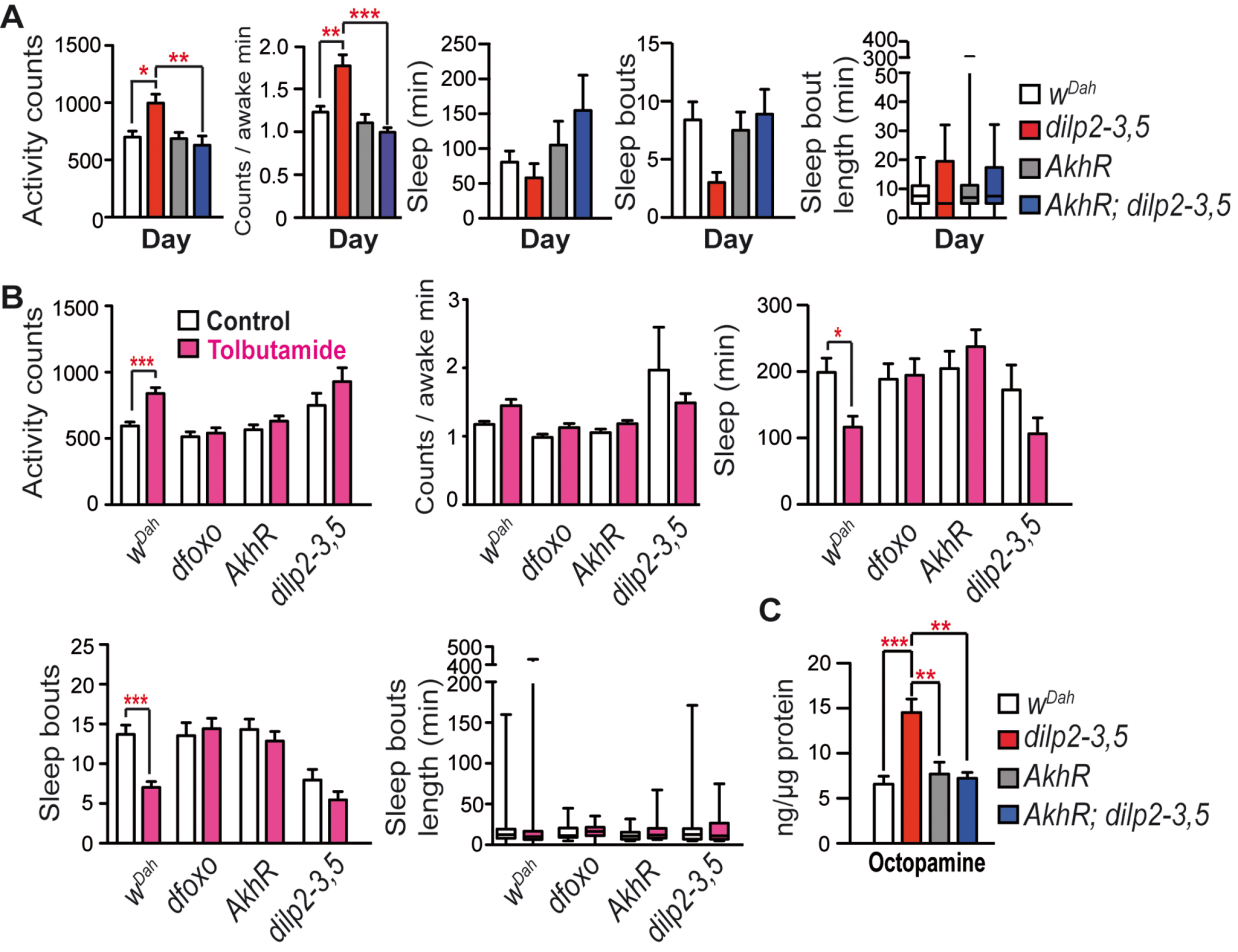
Day hyperactivity of IIS mutants is dependent on the *AkhR*. (A) Loss of *AkhR* abrogated the day activity phenotype of *dilp2-3,5* mutants (age 15 d, *w^Dah^ n* = 18, *dilp2-3,5 n* = 15, *AkhR n* = 18, *AkhR dilp-3,5 n* = 17). GLM was used to determine significance of genotype by genotype interactions in sleep and activity behaviours on loss of *AkhR* in controls and *dilp2-3,5* mutants. Significant differences were seen in day activity (*p* = 0.0057) and day bout number (*p* = 0.044) but not in day sleep (*p* = 0.14) or night behaviours (activity *p* = 0.09, sleep *p* = 0.63, bout number *p* = 0.22, night bout length *p* = 0.067). Corresponding nighttime behaviours are shown in Figure S4A. (B) Two-day tolbutamide (1.35 mg/ml) treatment increased day activity of *w^Dah^* flies. Lack of *dfoxo*, *AkhR*, or *dilp2-3,5* blocked the tolbutamide effect on day activity (nighttime behaviour shown in Figure S4B) (age 15 d, *w^Dah^ n* = 51/47, *AkhR n* = 30/34, *dfoxo^Δ94^ n* = 36/34, *dilp2-3,5 n* = 22/18, +/− tolbutamide). Analysis of genotype by treatment interactions (GLM) in sleep and activity behaviours on tolbutamide treatment in *IIS*, *AkhR*, and *dfoxo* mutants compared to controls showed day activity (*p* = 0.049), day sleep (*p* = <0.0001), and bout number (*p* = 0.008) were significantly different. However, no differences were seen in night behaviours (activity *p* = 0.58, sleep *p* = 0.89, bout number *p* = 0.28). (C) Mass spectrometry measurement of octopamine levels in head extracts (age 10 d, *w^Dah^ n* = 7, *dilp2-3,5 n* = 6, *AkhR n* = 6, *AkhR,dilp2-3,5 n* = 6). (A and B) Kruskal Wallis test with Dunn's multiple comparison test (selected pairs). (C) Mann–Whitney test. (C) One-way ANOVA with Bonferroni's multiple comparison test. ***p<0.001, **p<0.01, and *p<0.05. Error bars represent s.e.m.

To test the role of AKH in the day activity and sleep phenotypes of IIS mutants, we generated *dilp2-3,5* mutants lacking the *AkhR* receptor (CG11325). Loss of *AkhR* had no effect on day activity or sleep of wild-type flies, but it abrogated the increased day activity of IIS mutants, without affecting night activity, night sleep duration, or number of night sleep bouts (Figure 3A and Figure S4A). Tolbutamide increases AKH release through targeting the sulphonylurea receptor on AKH-producing cells [28]. In control flies, treatment with tolbutamide increased day activity, whilst reducing day sleep behaviours. In contrast, tolbutamide treatment did not increase day activity in *dilp2-3,5*, *dfoxo*, or *AkhR* mutants (Figure 3B and Figure S4B). Reduced IIS thus acts through increased AKH activity to induce its day activity and sleep phenotypes.

AKH increases activity in cockroaches through activation of the AkhR in octopaminergic cells [31], and we therefore hypothesised that reduced IIS might affect the level of the arousal-promoting, biogenic amine octopamine [32]. To determine if changes in the level of octopamine underlay the day activity and sleep phenotypes of IIS mutants, we used mass spectrometry to measure octopamine and found that levels were significantly increased in head extracts from IIS mutants (Figure 3C). Loss of *AkhR* abrogated the increased octopamine levels of *dilp2-3,5* mutants, suggesting that *AkhR* mediated the effect of reduced IIS on octopaminergic-signalling-mediated day activity (Figure 3C). To address the mechanism underlying this increase in octopamine levels, we examined the dopamine/octopamine biosynthetic pathway, but found expression of enzymes within this pathway to be unaltered (Figure S5A). However, tyramine, the precursor of octopamine, was significantly reduced (Figure S5B), suggesting increased enzymatic activity of tyramine β-hydroxylase in IIS mutants.

**Figure 4:**
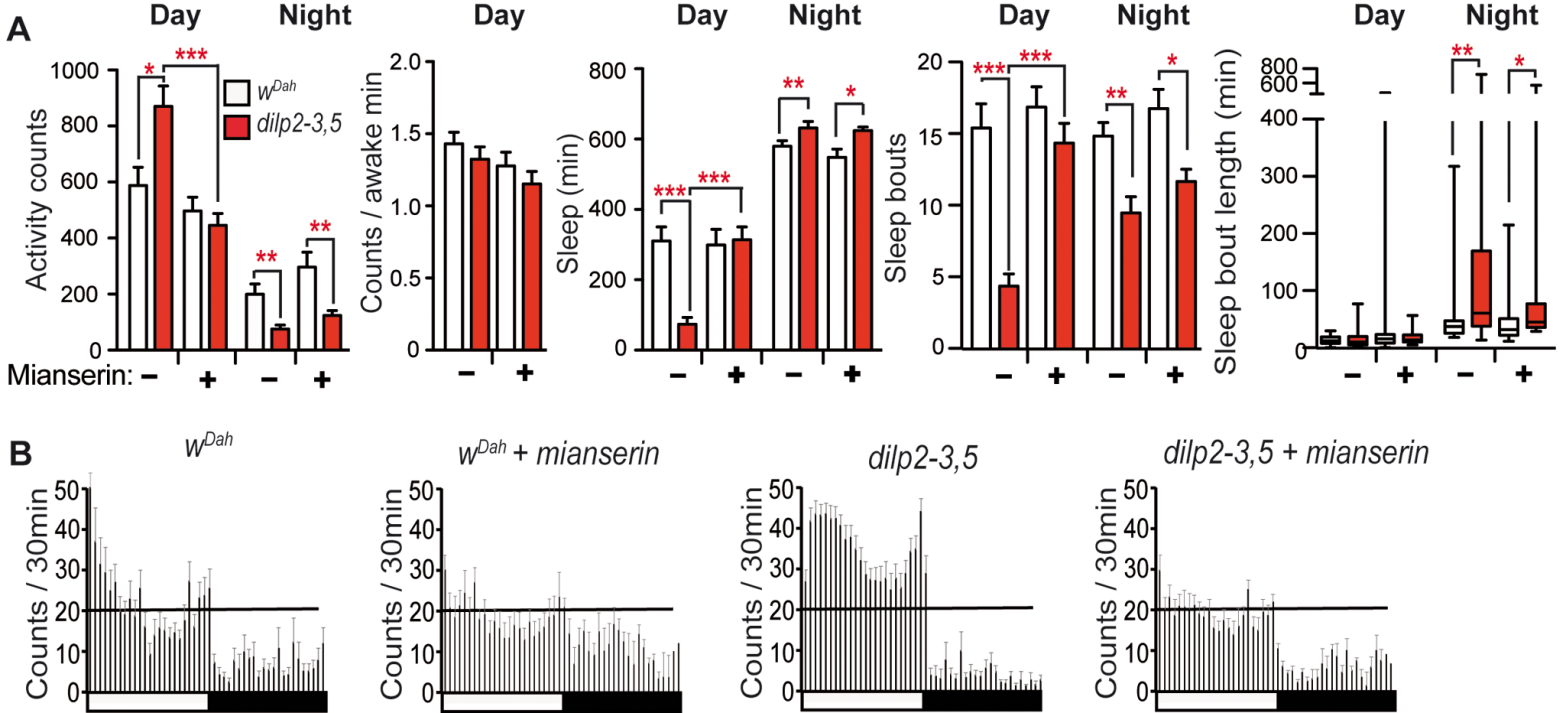
Reduced IIS causes day hyperactivity through increased octopaminergic signalling. (A) Two days feeding with mianserin hydrochloride (0.2 mg/ml) reverted the day activity phenotype of *dilp2-3,5* mutants (age 10 d), but not night activity, sleep, sleep bouts, and sleep bouts length (*w^Dah^ n = *17/24, *dilp2-3,5 n* = 17/27 +/− mianserin). GLM was used to determine significance of treatment by genotype interactions in sleep and activity behaviours on treatment with mianserin in controls and IIS mutants. Significant differences were seen in day activity (*p* = 0.0031), in day sleep (*p* = 0.0148), and day bout number (*p* = 0.002), but not in night behaviours (activity *p* = 0.31, sleep *p* = 0.49, bout number *p* = 0.72, night bout length *p* = 0.15). (B) Average activity count data (30 min bins) under 12∶12 h LD. (A) Kruskal Wallis test with Dunn's multiple comparison of selected pairs. ***p<0.001, **p<0.01, and *p<0.05. Error bars represent s.e.m.

To determine if increased day activity of IIS mutants was caused by increased octopaminergic signalling, we fed flies with mianserin hydrochloride, an inhibitor of octopaminergic signalling, which acts by inhibiting octopamine-induced cAMP increase [33]. Brief (2 d) feeding with mianserin hydrochloride did not affect activity or sleep of wild-type flies, but abrogated the increased day activity, sleep, and bout number but not the night sleep phenotypes of IIS mutants, indicating that reduced IIS induces activity through increased octopaminergic signalling (Figure 4A–B). However, enhanced octopaminergic signalling did not underlie longer lifespan of IIS mutants, since mianserin hydrochloride treatment of *dilp2-3,5* mutants did not affect their lifespan (Figure S5C), which also indicates that the chemical was not toxic to the flies.

Reduced IIS thus acts through reduced AKH and octopaminergic signalling to induce day activity and sleep phenotypes in *Drosophila*.

### Reduced TOR Activity Mediated the Effects of Reduced IIS on Night Activity and Sleep

**Figure 5:**
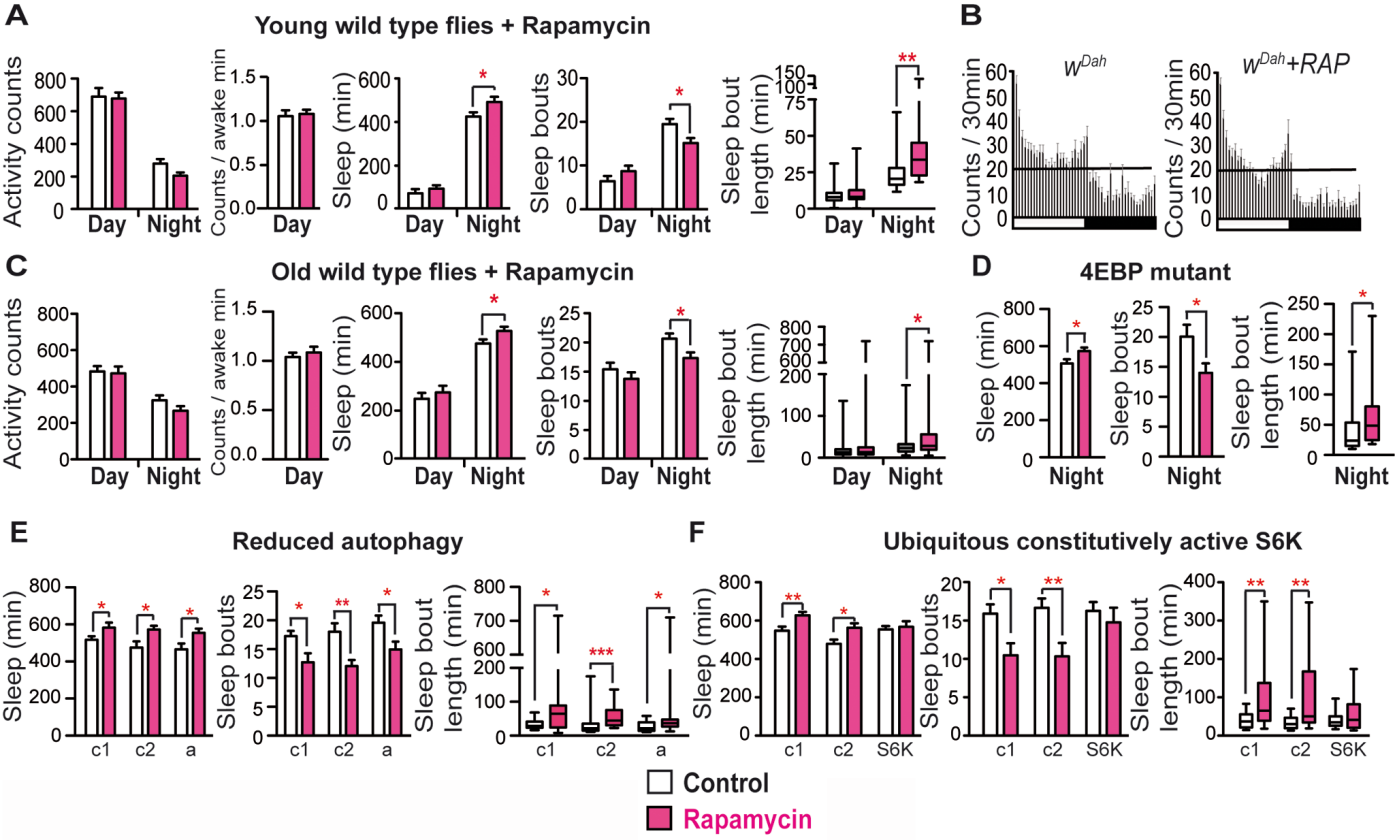
Rapamycin rescued age-related night sleep fragmentation in an S6K-dependent manner. (A) Rapamycin treatment (9 d) did not significantly affect day/night activity or wakefulness, but significantly increased night sleep duration, reduced night sleep fragmentation, and increased the length of night sleep periods (*w^Dah^*, age 10 d, *n* = 21/20 control/rapamycin). (B) Average activity count data (30 min bins) under 12∶12 h LD. (C) Acute rapamycin treatment of 45-d-old flies for 3 d did not significantly affect day/night activity or wakefulness, but significantly increased night sleep duration, reduced night sleep fragmentation, and increased the length of night sleep periods (*w^Dah^*, *n* = 64/64). Rapamycin-mediated night sleep, bout number, and bout length were independent of (D) 4E-BP (*n* = 19/19) and (E) reduced autophagy (a = *da-Gal4/UAS-ATG5-RNAi*, *n* = 20/17 or genetic controls c1 = *da-Gal4/+*, *n = *20/21 and c2 = *UAS-ATG5-RNAi/+*, *n* = 23/19). Flies with reduced autophagy responded to rapamycin as controls in sleep (*p* = 0.81), bout number (*p* = 0.82), and night bout length (*p* = 0.42) (GLM). (F) Ubiquitous expression of constitutively active S6K blocked the rescue of night sleep fragmentation by rapamycin (c1 = *da-Gal4/+*, *n = *20/21, and c2 = *UAS-S6K^STDETE^/+*, *n = *20/18, *S6K = da-Gal4/UAS-S6K^STDETE^*, *n = *20/17). Flies expressing a constitutively active form of S6K significantly differed from controls in the response to rapamycin (sleep *p* = 0.01, bout number *p* = 0.03, night bout length *p* = <0.0001, GLM). Kruskal Wallis test with Dunn's multiple comparisons of selected pairs. ***p<0.001, **p<0.01, and *p<0.05. Error bars represent s.e.m. Day behaviours of (D–F) are shown in Figure S6B–D.

IIS increases activity of the TOR kinase, which regulates translation through 4E-BP and S6 Kinase (S6K) and autophagy through *atg* genes [34]. TOR is inhibited by rapamycin, which also extends yeast, worm, fly, and mouse lifespan [16],[17],[35],[36]. Feeding wild-type flies with rapamycin increased night sleep duration and bout length and reduced number of sleep bouts, but did not affect day sleep, wakefulness, or activity (Figure 5A–B), suggesting that TOR might mediate the effects of reduced IIS on night sleep. In support of this hypothesis, rapamycin did not affect the night activity and sleep phenotypes of IIS mutants (Figure S6A). Night sleep fragmentation occurs during ageing, and to determine if acute treatment of old flies with rapamycin could ameliorate their sleep fragmentation, we treated 42-d-old flies over a brief, 3-d, period, which resulted in increased night sleep duration, fewer night sleep bouts, and increased bout length, without affecting day behaviours (Figure 5C). Reduced TOR signalling thus mediated the effects of reduced IIS on night activity and sleep, and acute inhibition with rapamycin could rescue the sleep fragmentation of ageing flies.

### S6K Mediated Effects of Reduced IIS on Night Sleep

Rapamycin-mediated lifespan extension in *Drosophila* is dependent on decreased S6K activity and increased autophagy, but not on 4E-BP [16],[17]. Neither loss of 4E-BP nor blocking autophagy counteracted the rescue of night sleep fragmentation by rapamycin (Figure 5D–E and Figure S6B–C). However, ubiquitous expression of constitutively active S6K did suppress the effect of rapamycin (Figure 5F and Figure S6D). These findings demonstrate that rapamycin acted through reduced S6K activity to rescue night sleep fragmentation.

TOR up-regulates translation [13], and inhibition of protein synthesis through cycloheximide administration enhances sleep in mammals [37]. To probe a possible role of reduced protein synthesis in consolidation of sleep in flies, we fed them cycloheximide. This resulted in increased day and night sleep duration and bout length and reduced number of sleep bouts, and in reduced day and night activity (Figure S7A), suggesting that inhibition of protein synthesis increases sleep in flies, and could account for the effects of TOR and S6K on night sleep. Prolonged cycloheximide feeding (5 d) did not increase mortality (unpublished data), and nor did it further affect activity or sleep patterns, but we cannot exclude the possibility that reduced activity of the flies was a toxic side-effect of the drug (Figure S7B). These findings suggest a role of reduced protein synthesis in the night sleep phenotypes from reduced IIS and TOR activity.

### Reduced IIS Altered Dopamine Receptor Expression

**Figure 6:**
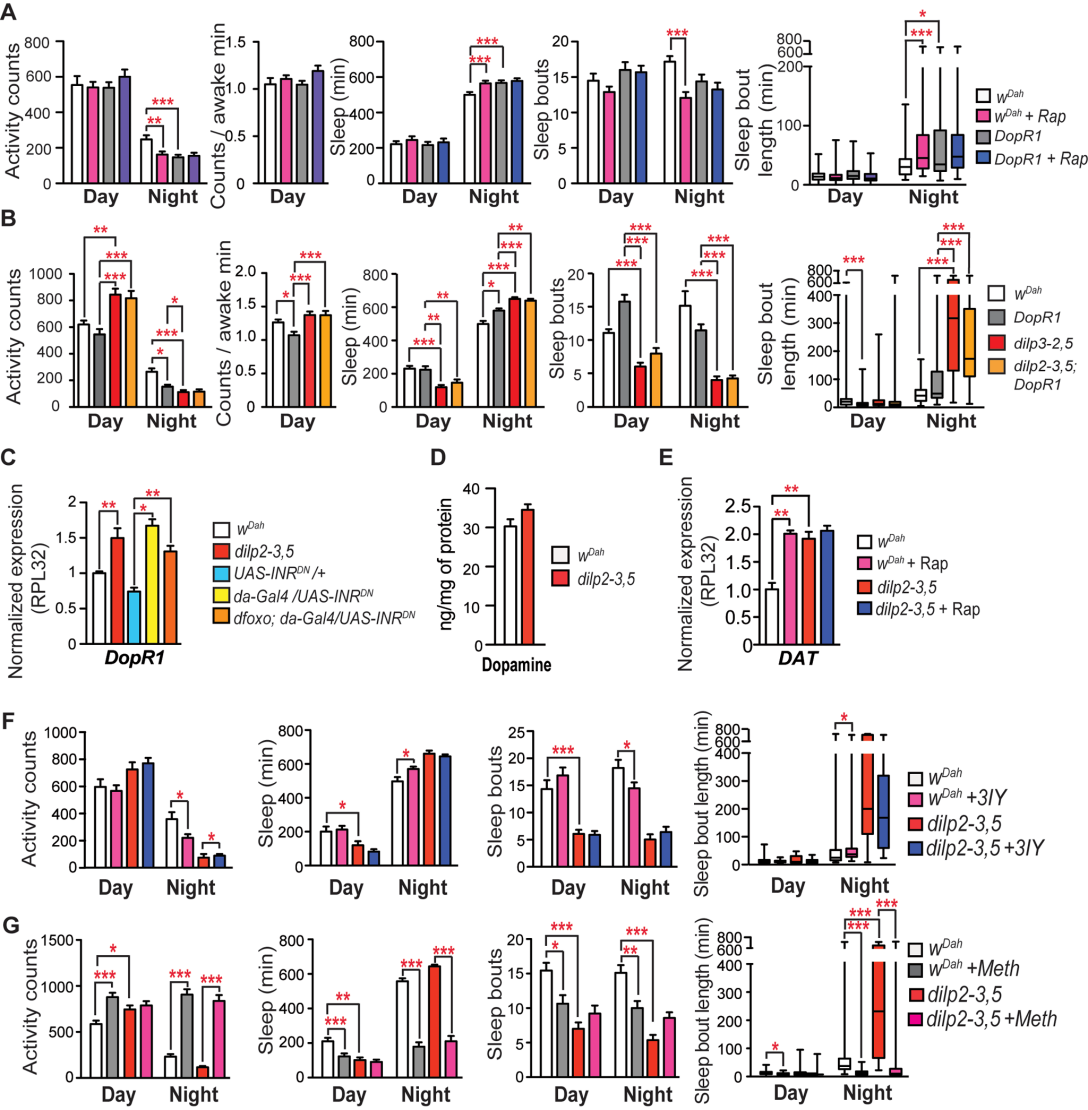
Dopamine receptor mutants do not respond to rapamycin treatment. (A) *DopR1* mutants had similar activity and sleep features as rapamycin-fed flies. Behaviour of *DopR1* mutants was not affected by rapamycin feeding (age 10 d, *n* = 64 for all genotypes). Flies were fed with rapamycin for 9 d. (B) *dilp2-3,5*, *DopR1* mutants had similar activity and sleep features as *dilp2-3,5* mutants (*n = *64 for all genotypes). (C) QRT-PCR analysis of dopamine receptor (*DopR1*) expression normalized to *Rpl32* expression and controls in head extracts of *dilp2-3,5* mutants (age 10 d, *n* = 9) and *da-Gal4/UAS/INR^DN^* flies and *da-Gal4/INR^DN^;dfoxo* mutants (age 10 d, *n* = 3). (D) Mass spectrometry measurement of dopamine levels in head extracts of female flies (age 10 d, *n = *3). (E) QRT-PCR analysis of *DAT* expression, normalized to *Rpl32* expression (*n* = 9). (F) Behaviour of IIS mutants after short-term exposure (2 d) to the tyrosine hydroxylase inhibitor 3IY (5 mg/ml) (age 35 d, *n* = 32 for all genotypes). IIS mutants differed from controls in the nighttime activity and sleep response to 3IY treatment, but not in bout or bout length (activity *p* = 0.044, sleep *p* = 0.014, bout number *p* = 0.276, night bout length *p* = 0.463, GLM). (G) Behaviour of IIS mutants after short-term (12 h) exposure to METH (1 mg/ml) (age 25 d, *n* = 48 for all genotypes). IIS mutants differed from controls in daytime behaviours after METH treatment (activity *p* = 0.005, sleep *p* = <0.0001, bout number *p* = 0.0002, bout length *p* = 0.031, GLM) along with nighttime bouts (*p = *<0.0001) and bout length (*p = *<0.0001), whereas nighttime activity and sleep did not differ (activity *p = *0.796, sleep *p = *0.352, GLM). (A, B, G) Kruskal Wallis test with Dunn's multiple comparison of selected pairs. (F) Individual comparisons by Mann–Whitney U test. (C–E) Two-tailed *t* test. ***p<0.001, **p<0.01, and *p<0.05. Error bars represent s.e.m. (C, E, F) QRT-PCR analysis normalized to *RNApolII* expression shown in Figure S5D.

Dopaminergic signalling regulates sleep in flies [3],[4], and disruption of the dopamine receptor 1 gene (*DopR1*) decreases night activity, increases night sleep, and decreases night sleep fragmentation [38], phenotypes that we also observed in the present study (Figure 6A). We therefore investigated the role of dopaminergic signalling in mediating the effects of rapamycin on night sleep phenotypes, by administration of rapamycin to *DopR1* flies (Figure 6A). In contrast to the effects in wild-type controls, rapamycin did not affect night activity, night sleep, night sleep bout number, or bout duration of *DopR1* mutants (Figure 6A). GLM indicated that response of *DopR1* mutants to rapamycin was significantly different from that of wild-type flies for night activity (*p = <0.0001*), night bout number (*p = 0.025*), and night bout length (*p = 0.015*), but not for night sleep (*p = 0.57*). Furthermore, *dilp2-3,5;DopR1* double mutants did not differ in their activity and sleep phenotypes from *dilp2-3,5* mutants (Figure 6B). These results indicate that reduced dopaminergic signalling mediates the effects of reduced IIS on night sleep activity and sleep phenotypes. We therefore investigated the mechanisms mediating the effects of reduced IIS and TOR activity on dopaminergic signalling.

We first tested whether the night phenotypes of the IIS mutants were a consequence of decreased DopR levels, by quantifying *DopR1* transcript levels in *dilp2-3,5* and *INR^DN^* flies. Surprisingly, transcript levels of *DopR1* were increased in both *dilp2-3,5* mutants and *INR^DN^* flies, and also in *INR^DN^* flies lacking *dfoxo* (Figure 6C), suggesting a compensatory response of dopamine receptor expression to a different lesion in dopaminergic signalling.

We next determined whether overall dopamine levels in the flies were affected by reduced IIS. We quantified dopamine synthesis in *dilp2-3,5* mutants but found no difference in either the expression levels of dopamine biosynthetic enzymes (Figure S5A) or in total dopamine (Figure 6D). However, after treatment with rapamycin or reduced IIS, expression levels of dopamine transporter (DAT) were increased (Figure 6E). Under normal physiological conditions, dopamine signalling is regulated by the level of extracellular dopamine and the rate of DAT-mediated clearance of dopamine from the synaptic cleft. Increased DAT expression in *Drosophila* IIS mutants could therefore blunt dopaminergic signalling and induce the compensatory changes seen elsewhere in the pathway.

If increased DAT activity in IIS mutants does indeed regulate their night sleep phenotypes, then these should be disrupted by pharmacological inhibition or overactivation of dopaminergic signalling. 3-Iodo-L-tyrosine (3IY) inhibits dopaminergic signalling by inhibiting tyrosine hydroxylase, the rate-limiting enzyme in the synthesis of dopamine, and hence reducing dopamine levels. If the nighttime behaviours of IIS mutants are mediated through increased DAT activity, and therefore reduced dopaminergic signalling, short-term treatment with 3IY should not affect IIS mutant behaviours, whereas controls should show behaviours associated with reduced dopaminergic signalling. Although 3IY treatment had no effect on day behaviours of wild-type or IIS mutant flies, in wild-type flies nighttime activity was reduced and sleep increased, with fewer, longer sleep bouts (Figure 6F). In contrast, IIS mutants showed no response to 3IY at night apart from a slight increase in activity, the opposite effect to that seen in controls (Figure 6F).

Methamphetamine (METH) increases dopaminergic signalling, by preventing dopamine clearance from the synaptic cleft by DAT and increasing dopamine efflux through DAT from the presynaptic cells. METH would therefore be predicted to revert the night activity and sleep phenotypes of IIS mutants towards those of controls. In accordance with the results of previous studies [3], short-term exposure (12 h) to METH increased both day and night activity in wild-type flies, decreased their day and night sleep, and reduced their day and night sleep bouts and sleep bout length (Figure 6G). In contrast, METH treatment had no effect on the day phenotypes of *dilp2-3,5* mutants, whereas it significantly increased their night activity and reduced their night sleep and sleep bout length to levels similar to those of treated controls (Figure 6G). Night sleep bout number and bout length of *dilp2-3,5* mutants increased significantly (GLM, *p* = <0.0001) more than that of controls in response to METH treatment. Night sleep phenotypes of the IIS mutants were thus more responsive to METH treatment, as predicted if reduced IIS increases DAT activity. Taken together, these results indicate that reduced IIS induced night activity and sleep phenotypes by modulating DAT activity.

## Discussion

### IIS Signalling and Sleep

Sleep syndromes are highly prevalent in elderly humans and, with a continuing increase in life expectancy and a greater proportion of elderly people worldwide, effective treatments with fewer side effects are becoming increasingly needed. Sleep in flies shares striking similarities with sleep in humans [26], including an age-related reduction in sleep quality [2]. Here, we used *Drosophila* to examine age-related sleep pathologies and to suppress these pathologies through genetic and pharmacological perturbation of insulin/IGF and TOR signalling.

We showed that the highly conserved IIS pathway, with roles in growth and development, metabolism, fecundity, stress resistance, and lifespan, also affects sleep patterns in *Drosophila*. Reduced IIS increases and consolidates night sleep, while decreasing day sleep and inducing day activity. Interestingly, *dilp2-3* double mutant flies as well as flies with neuron or fat-body-specific down-regulation of IIS showed no obvious or only mild sleep phenotypes in a previous study [39], suggesting that a strong and/or systemic reduction in IIS activity may be necessary to induce the activity and sleep phenotypes. Consistently, *dilp2-3* double mutant flies have very mild growth, lifespan, and metabolic phenotypes compared to the *dilp2-3,5* triple mutant flies used in this study [21]. Reduced IIS activity resulted in increased sleep consolidation in young flies. Importantly, reduced IIS ameliorated the age-related decline in sleep consolidation seen in wild-type flies, thus showing that it is malleable. Contrary to the increased sleep consolidation with reduced IIS, high calorie diets have been reported to accelerate sleep fragmentation [40]. Furthermore, dietary sugar affects sleep pattern in flies [41]. Taken together, these findings reveal a role of nutrition and metabolism in sleep regulation and age-related sleep decline in flies.

In humans, several studies suggest a link between nutrition and sleep. The amino acid tryptophan can promote sleep, possibly by affecting synthesis of the sleep regulators serotonin and melatonin. Also, the carbohydrate/fat content of the diet seemingly affects sleep parameters. However, most of these studies are based on correlational methods and small sample size, and it is not yet clear how diet affects sleep [42]. Interestingly, sleep duration can affect metabolism, risk for obesity and diabetes, and even food preference [43],[44]. These findings associate sleep and metabolism; thus, manipulation of nutrient-sensing pathways, such as IIS and TOR signalling, may affect activity and sleep in humans.

### Reduced IIS Induces Day Hyperactivity through dFOXO and Octopamine

The transcription factor FoxO is an important downstream mediator of IIS. In *C. elegans* all aspects of IIS are dependent on *daf-16*, the worm ortholog of *foxO* [9]. In contrast, in *Drosophila* IIS-mediated lifespan extension is dependent on *dfoxo*, whereas several phenotypes of reduced IIS are *dfoxo-*independent [12]. Activity and sleep were unaffected by the loss of *dfoxo* in wild-type flies. In contrast, under low IIS conditions loss of *dfoxo* specifically affected daytime behaviour, with night time behaviours unaffected. Reduced IIS therefore affects day and night sleep and activity through distinct mechanisms. It also uncouples the effects of IIS on lifespan and on night sleep consolidation, since *dfoxo* is essential for extended longevity of flies with reduced IIS. dFOXO has been previously shown to increase neuronal excitability, possibly via transcription of ion channel subunits or other regulators [45].

We suggest that a possible such regulator could be octopaminergic signalling, known to promote arousal in *Drosophila* [32],[46]. Octopamine, the arthropod equivalent of noradrenaline, regulates several behavioural/physiological processes, including glycogenolysis and fat metabolism, as well as synaptic and behavioural plasticity [47]. Moreover, octopamine can affect sleep by acting on insulin-producing cells in the fly brain, thus linking IIS and sleep/activity [46]. Indeed, we found that IIS mutants have increased octopamine levels and, importantly, pharmacological inhibition of octopaminergic signalling reverted the increased day activity of IIS mutants. Noteworthy, mRNA expression of octopamine biosynthetic enzymes was not changed, but tyramine levels were significantly reduced, suggesting that increased translation, reduced degradation, or increased activity of the tyramine-β hydroxylase regulates octopamine levels in IIS mutants. In contrast to day activity, increased lifespan of IIS mutants was not affected by pharmacological inhibition of octopaminergic signalling, thus separating longevity from the day activity phenotype.

### IIS Regulates Octopaminergic Signalling through AKH

The effect of reduced IIS on day sleep/activity was mediated through AKH, the equivalent of human glucagon, an antagonist of insulin [28],[29],[48]. In flies, AKH coordinates the response to hunger through mobilizing energy stores and increasing food intake [49], as well as inducing a starvation-like hyperactivity [30],[48]. Loss of AKH receptor (*AkhR*) abrogated the increased activity of IIS mutants without affecting night sleep. These results demonstrate that day and night phenotypes of IIS mutants can be uncoupled, suggesting that the increased night sleep of IIS mutants is not just a compensatory consequence of increased day activity.

*dilp2-3,5* mutants have increased octopamine levels, and loss of *AkhR* in the *dilp2-3,5* mutant background reduced their octopamine level back to wild-type levels, suggesting that *AkhR*-mediated regulation of octopamine controls day hyperactivity in IIS mutants. In support of our findings, octopaminergic cells mediate the increased activity effect of AKH in other insects [31]. Flies lacking dFOXO did not respond to chemically induced AKH release, suggesting that AKH affects activity through dFOXO. Therefore, we suggest that dFOXO and *AkhR* act through overlapping mechanisms to enhance octopaminergic signalling and induce activity.

In flies, *AkhR* is highly expressed in fat body and its loss alters lipid and carbohydrate store levels [50]. Therefore, *AkhR* might indirectly enhance octopaminergic signalling through alterations in lipid and carbohydrate metabolism. In support of this idea, lipid metabolism affects sleep homeostasis in flies [51]. Additionally, *AkhR* expression in octopaminergic cells could regulate octopamine synthesis and release in flies [31]. Interestingly, expression of *AkhR* is altered in *dfoxo* mutants [52], thus implicating dFOXO in *AkhR* regulation. Both are highly expressed in fat body, an important organ for metabolism in flies, and fat-body-specific insulin receptor may regulate *AkhR* function through dFOXO activation.

In larval motor neurons, dFOXO increases neuronal excitability [45] and octopamine increases glutamate release, suggesting there is at least a spatial functional link between the two [47],[53]. Thus, together with a possible role in *AkhR* synthesis, dFOXO could act downstream of octopamine to increase activity.

### IIS Regulates Night Sleep through TOR

To determine the mechanism underlying the IIS-dependent amelioration of age-related sleep decline, we investigated downstream components and genetic interactors of IIS. One such interactor that affects health and ageing is TORC1. TORC1 is a major regulator of translation, through S6K, 4E-BP, and of autophagy, through ATG1 [14],[15]. Inhibiting TOR signalling, and thus translation, by rapamycin treatment in wild-type flies recapitulated the sleep features of IIS mutants, even in old flies. This rescue of sleep quality was blocked by ubiquitous expression of activated S6K, suggesting that reduced S6K activity is required for the rescue. Our findings, together with previous results showing S6K to regulate hunger-driven behaviours, highlight the importance of S6K as a regulator of behaviour in flies [54]. Thus, manipulating TOR signalling can improve sleep quality through S6K.

In mammals, rapamycin treatment has beneficial effects on behaviour throughout lifespan. Although complete block of TOR activity is detrimental for long-term memory [55], a moderate decrease through rapamycin treatment can improve cognitive function, abrogate age-related cognitive deterioration, and reduce anxiety and depression [56]. Moreover, increased TOR activity throughout development is detrimental for neuronal plasticity and memory [56]. In flies, rapamycin prevents dopaminergic neuron loss in mutants with parkinsonism [57]. Although the role of TOR in brain function has not been well studied in flies, the advantageous effect of rapamycin in both mammalian brain function and sleep in flies may be mediated through common neurophysiological mechanisms.

Gene expression studies have suggested that protein synthesis is up-regulated during sleep [2],[58], which may be an essential stage in macromolecular biosynthesis [59]–[61]. Consistent with this, inhibiting protein synthesis in specific brain domains prolongs sleep duration in mammals, suggesting that sleep is maintained until specific levels of biosynthesis occur and aids in explaining the ubiquitously conserved need for sleep [37],[62]. Here, brief cycloheximide treatment prolonged night sleep and increased consolidation in flies, indicating an evolutionarily conserved role for protein synthesis inhibition on sleep regulation. Contrary to reduced IIS, cycloheximide reduced day activity, possibly due to the global effect of cycloheximide on protein synthesis or due to toxic defects in flies' physiology. Decreased protein synthesis rates may enhance the necessity for increased sleep duration, to allow sufficient synthesis of proteins and other macromolecules during sleep, allowing organisms to be healthy and functional during the day.

Alternatively, the effect of protein synthesis inhibition on night sleep could be the result of reduced expression of specific sleep regulators. We found that *DopR1* and *dilp2-3,*5 mutants share night phenotypes and that rapamycin did not affect sleep of *DopR1* mutants, suggesting that TOR acts on dopaminergic signalling to affect night sleep. Reduced IIS elevated expression of *DopR1*, independently of dFOXO, in accordance with data from mammals [63]. This effect may be feedback caused by down-regulation of dopaminergic signalling in IIS mutants, although not through direct regulation of DopR. Under normal physiological conditions, dopamine signalling is determined by the level of extracellular dopamine and the rate of DAT-mediated dopamine clearance from the synaptic cleft [64]. The rate of dopamine clearance is dependent on the turnover rate of DAT and the number of functional transporters at the plasma membrane [65]–[67]. We found that reduced IIS and rapamycin treatment induced increased expression of DAT, suggesting an increased rate of dopamine clearance from the synaptic cleft, and thus a reduction in the amplitude of dopamine signalling, without changes in total dopamine levels. DAT function and IIS have recently been linked in mammals. DAT function increases upon insulin stimulation and is diminished on insulin depletion, through alterations in DAT membrane localization [68]. However, IIS-dependent regulation of DAT subcellular localization in *Drosophila* has not yet been demonstrated. Our data suggest down-regulating dopaminergic signalling, either by loss of DopR1 or increasing DAT levels, is beneficial for sleep quality. In agreement with this we show that artificially increasing dopaminergic signalling, through short-term METH treatment, increases both day and night activity and reduces night sleep, and reverts the beneficial effect of reduced IIS on night behaviours. In mammals, cocaine administration, which enhances dopaminergic signalling, increases TOR activity. Also, rapamycin blocks cocaine-induced locomotor sensitization [69]. Interestingly, cocaine stimulates S6K phosphorylation in rat brains, and this effect is blocked by rapamycin. Taken together, these results show that in flies and mammals dopaminergic and IIS/TOR signalling may interact in similar ways.

### Conclusions

In conclusion, reduced IIS extends lifespan in diverse organisms. Here we have shown that it can also ameliorate age-related sleep fragmentation, but that the mechanisms by which it does so are distinct from those by which it extends lifespan. Reduced IIS affected day activity and sleep phenotypes through increased octopaminergic signalling, but enhanced octopaminergic signalling did not increase lifespan. Similarly, in *Drosophila* increased lifespan from reduced IIS requires *dfoxo*, but the night sleep phenotypes of IIS mutants were independent of this transcription factor. Reduced IIS thus acts through multiple pathways to ameliorate different aspects of loss of function during ageing. IIS links metabolism and behaviour through its components, such as S6K and dFOXO, which act through different neuronal circuits and neurons to affect sleep (Figure S8, including interactions shown in [45],[53]). The strong evolutionary conservation of these circuits and their functions suggests that pharmacological manipulation of IIS effectors could be beneficial in treatments of sleep syndromes in humans.

## Materials and Methods

### Fly Stocks and Fly Husbandry

All mutant chromosomes and transgenes were backcrossed into a *white* Dahomey (*w^Dah^*) wild-type strain for at least eight generations. Fly stocks were kept at 25°C on a 12 h light and 12 h dark cycle (12∶12h LD) and were fed a standard sugar/yeast/agar diet (SYA) [70]. In all experiments we used virgin females that were reared at controlled larval densities. Adult flies were kept in SYA food vials (10 to 30 flies per vial) prior to behavioural analysis. During activity recording, flies were fed with SYA food. The daughterless-Gal4 (da-Gal4) driver and the lines UAS-dInR^A1409K^ and UAS-S6K^STDETE^ were obtained from the Bloomington Drosophila Stock Center (Bloomington, IN). The *dfoxo^Δ94^* mutant was kindly provided by Cathy Slack [12]. The *atg5* RNAi was kindly provided by Thomas P. Neufeld [71]. 4E-BP and *AkhR* null mutants [72] were kindly provided by Paul F. Lasco and Ronald P. Kühnlein, respectively. *DopR1* mutants were kindly provided by David Anderson [38].

### Circadian, Sleep, and Activity Analysis

For circadian rhythm analysis, 20-d-old virgin females were placed in an activity monitoring system (DAM2, Trikinetics) and were entrained in 12∶12 h LD conditions for 5 d prior to a 5-d period of activity recordings under 12∶12 h DD (free-running condition). During the 10 d of recording, flies were kept continuously in the activity monitoring system. Activity recordings were collected in 30 min intervals and rhythmicity of individual flies was analysed using the MAZ package. Periodicity was calculated using the autocorrelation method [73]. As a measure of significance for the rhythmic components, we used a Monte Carlo approach [73].

For sleep analysis, virgin females were kept under 12∶12 h LD conditions for 5 d in the activity monitoring system. Activity was measured in 5 min intervals, and data from the third day were analysed in Excel. Sleep was defined as an interval of 5 min or more of nonactivity. Wakefulness, the average activity per waking minute, was calculated as previously described [24]. For analysis of aged flies (Figure 1 and Figure S2), we used flies from the same collection, kept with SYA food vials throughout their life. Sleep and activity of randomly selected individuals, at different ages, were recorded for 5 d in the activity monitoring system. All behavioural data (activity, sleep duration, wakefulness, sleep bout number, and sleep bout length) are represented by mean values.

### Biogenic Amine Measurements

Amine levels in virgin females were measured with mass spectrometry. Fly head extracts were homogenised in 0.1% formic acid and filtered (0.45 µm) at 13,000 g for 10 min (4°C). The filtrate was transferred into total recovery vials (Waters, Milford, MA) and immediately frozen at −20°C. Directly before analysis, samples were thawed.

For absolute quantification of Dopamine, Octopamine, and Tyramine in positive ESI MRM (multireaction monitoring) mode, an Acquitiy UPLC/Xevo TQ (Waters) with MassLynx and absolute quantification TargetLynx (Waters) were used. An Acquity UPLC BEH C18 1.7 µm, 2.1×50 mm column was used at 25°C. Solvent A was 0.1% formic acid (Biosolve) and B acetonitrile (Biosolve). A linear gradient from 99% A to 0% in 2 min at a flow rate of 0.35 ml/min was used. The following MRM transitions were used: for Dopamine m/z 154.01 (M+H^+^)^+^ to 91.04 (quantifier) collision energy 22V, cone 12V; for d4-Dopamine m/z 158.03 to 94.79 (quantifier) collision 22V, cone 8V; for Octopamine m/z 154.03 to 91.01 (quantifier) collision energy 18V, cone 8V; for d3-Octopamine m/z 156.97 to 93.07 (quantifier) collision 22V, cone was 17V; and for Tyramine m/z 138.03 to 76.95 (quantifier) collision 24V, cone 26V. Compounds were dissolved in 0.1% formic acid. Calibration curves were calculated with internal standards (d4-Dopamine 100 ng/ml, and d3-Octopamin 40 ng/ml), and for Tyramine an external calibration curve was calculated using the following concentrations: 0.5, 1, 2, 5, 10, 15, 25, 50, 100, 200 ng/mL. Correlation coefficient, r<0.990; response type, internal and external standard, area; curve type linear; weighting 1/x. The peak integrations were corrected manually, if necessary.

### Quantitative RT-PCR

Total RNA was extracted using Trizol (Invitrogen Corp.) according to the manufacturer's instructions and included a DNase treatment. SuperScript III first strand synthesis kit (Invitrogen Corp.) was used to prepare cDNA. Quantitative real-time PCR was performed with TaqMan primers (Applied Biosystems) in a 7900HT real-time PCR system (Applied Biosystems). Relative expression (fold induction) was calculated using the ΔΔC_T_ method and Rpl32 or *RNA pol II* as normalisation control.

### Chemicals

Rapamycin (LC Laboratories) was dissolved in ethanol and added to SYA food at a final concentration of 400 µM [17]. Tolbutamide (Santa Cruz Biotechnology) was dissolved in ethanol and added to SYA food at a final concentration of 1.35 mg/ml [74]. Cycloheximide and mianserin hydrochloride (Sigma) were dissolved in water and added to SYA food at final concentrations of 17 mM [75] and 0.2 mg/ml [32], respectively. METH hydrochloride (Sigma) was dissolved in ethanol and added to SYA food at a final concentration of 1 mg/ml [3],[4]. 3IY was added directly to SYA food at a final concentration of 5 mg/ml [3]. An overview of pharmacological sites of action can be found in Figure S8.

### Statistical Analysis

Statistical analysis was performed using GraphPad Prism 5.03 (GraphPad Prism Software, Inc) and JMP (v.10, SAS institute). For behavioural analysis we used the Kruskal Wallis with Dunn's multiple comparison test, for multiple comparisons, and Mann–Whitney test, for pair-wise comparisons. GLM was used to determine significance of genotype by treatment or age interactions on normalized activity and sleep behaviour data (where necessary data were transformed to reach normality criteria).

## Supporting Information

Figure S1 Altered activity and sleep patterns in IIS mutants. (A) Activity over 10 d (2 d per horizontal line) of 10-d-old *da-Gal4/UAS-INR^DN^* flies and controls under 12∶12 h LD (indicated by white and black bars) and 12∶12 h DD (*n* = 11 for all genotypes). Mean free run period (τ) in DD ± s.e.m. (B) Average activity count data (30 min bins) over a 24-h cycle under 12∶12 h LD (indicated by white and black bars) for *da-Gal4/UAS-INR^DN^* flies and controls (*n = *23 for all genotypes). (C) *da-Gal4/UAS-INR^DN^* flies were more active by day and less active at night, (D) slept less or the same during the day and more during the night, (E) had fewer night sleep bouts, and (F) significantly increased night sleep bout duration, compared to control flies. (G) Control flies, but not *da-Gal4/UAS-INR^DN^*, showed a significant age-related increase in night sleep bouts (ages 10 d, 20 d, 25 d, and 55 d). (C–G) *n = da-Gal4/+*, *n* = 28, 32, 24, and 29 for ages 10 d, 20 d, 25 d, and 55 d, respectively; *UAS-INR^DN^/+ n* = 25, 31, 23, and 23 for ages 10 d, 20 d, 25 d, and 55 d, respectively; and *da-Gal4/UAS-INR^DN^ n* = 27, 30, 31, and 49 for ages 10 d, 20 d, 25 d, and 55 d, respectively. Kruskal Wallis test with Dunn's multiple comparison test (selected pairs). ***p<0.001, **p<0.01, and *p<0.05. Error bars represent s.e.m.
(TIF)

Figure S2 Effect of light on activity and sleep of *dilp2-3,5* mutants. Activity and sleep of *dilp2-3,5* mutants and controls under 12∶12 hLD and 12∶12 h dark∶dark (DD) conditions. (A) Increased day activity of *dilp2-3,5* mutants was dependent on light and was lost under DD conditions. (B) In contrast to controls, which had reduced day sleep in DD conditions, *dilp2-3,5* mutants had increased day sleep in DD. (C) Sleep bout number was not affected by DD in *dilp2-3,5* mutants, but was decreased in controls. (D) *dilp2-3,5* mutants, but not controls, had increased day sleep bout length in DD. GLM showed that *dilp2-3,5* mutants had a significantly different response to light conditions in day activity (*p* = 0.0007), day sleep (*p* = <0.0001), day bout number (*p* = 0.0002), and day bout length (*p* = <0.0001) compared to controls. In contrast, no significant differences were seen in night behaviours (activity *p* = 0.1014, sleep *p* = 0.682, bout number *p* = 0.2019, bout length *p* = 0.7425). Data represent two independent experiments that were pooled: *w^Dah^ n = *126, *dilp2-3,5 n = *120. Ten-day-old flies, LD; 13-d-old, DD. Kruskal Wallis test with Dunn's multiple comparison test (selected pairs). ***p<0.001, **p<0.01, and *p<0.05. Error bars represent s.e.m.
(TIF)

Figure S3 Loss of *dfoxo* affected daytime activity and sleep phenotypes of *INR^DN^* flies. Independent experiment verifying activity and sleep phenotypes in Figure 2 (age 10 d). *dfoxo* indicates the *dfoxo^Δ94^* allele (*w^Dah^ n = *30, *dfoxo n = *27, *UAS-INR^DN^/+;dfoxo/+ n = *22, *da-Gal4/+;dfoxo/+ n = *32, *da-Gal4/UAS-INR^DN^;dfoxo n = *24, *da-Gal4 n = *27). Kruskal Wallis test with Dunn's multiple comparison test (selected pairs). ***p<0.001, **p<0.01, and *p<0.05. Error bars represent s.e.m.
(TIF)

Figure S4 Day hyperactivity, not nighttime behaviours, of IIS mutants is mediated through *AkhR*. (A) Nighttime behaviour data of Figure 3A. (B) Nighttime behaviour data of Tolbutamide treated flies in Figure 3B. Kruskal Wallis test with Dunn's multiple comparison test (selected pairs). ***p<0.001, **p<0.01, and *p<0.05. Error bars represent s.e.m.
(TIF)

Figure S5 Bioamine biosynthetic enzyme expression level is independent of IIS. (A) Bioamine biosynthetic pathways and QRT-PCR analysis of biosynthetic enzyme expression in *dilp2-3,5* mutant heads compared to controls (age 10 d), normalized to *Rpl32* (*n* = 6) or *RNA pol II* (*n* = 3) expression. (B) Mass spectrometry measurement of tyramine levels in head extracts (age 35 d, *w^Dah^ n* = 7, *dilp2-3,5 n* = 8). (C) Survival analysis of *w^Dah^* and dilp2-3,5 treated with mianserin (0.2 mg/ml) or control food. Significance determined by Log-rank test (*n = *100 for all genotypes/treatments). (D) QRT-PCR analysis normalized to *RNApolII* expression matching dataset in Figure 6C and E. (E) QRT-PCR analysis of *DAT* expression in aged *dilp2-3,5* mutant heads compared to controls, normalized to 10-d-old controls and *Rpl32* (*n* = 3) expression.
(TIF)

Figure S6 Effect of rapamycin on daytime behaviour of IIS/TOR signalling components. (A) Chronic rapamycin treatment (9 d) does not affect activity and sleep of IIS mutants (age 10 d, *n = w^Dah^* control/rapamycin 21/23, *dilp2-3,5* control/rapamycin 22/21), (B) *4EBP* mutants (*n = *19/19), (C) flies with reduced autophagy (*da-Gal4/UAS-ATG5-RNAi* (a) (*n = *20/17)) or genetic controls (*da-Gal4/+* (c1) (*n = *20/21) and *UAS-ATG5-RNAi/+* (c2) *n = *23/19), (D) flies ubiquitously expressing constitutively active S6K (*da-Gal4/UAS-S6K^STDETE^* (S6K) *n = *20/17) or genetic controls (*da-Gal4/+* (c1), *n = *20/21, and *UAS-S6K^STDETE^/+* (c2), *n = *20/18). Kruskal Wallis test with Dunn's multiple comparison test (selected pairs). ***p<0.001, **p<0.01, and *p<0.05. Error bars represent s.e.m. (Daytime behaviours from Figure 5D–F).
(TIF)

Figure S7 Cycloheximide affected both day and night behaviour. (A) Cycloheximide (CHX) treatment (17 mM) reduced day activity and increased day sleep but not wakefulness or day sleep bouts (age 10 d, *n* = 15/15 control/CHX treated). (B) Extended CHX treatment (5 d) did not alter behavior beyond that of 2 d treatment (*n = *control 2/5-d-old 42/43, CHX treated 2/5-d-old 29/28). Kruskal Wallis test with Dunn's multiple comparison test (selected pairs). ***p<0.001, **p<0.01, and *p<0.05. Error bars represent s.e.m.
(TIF)

Figure S8 Model depicting IIS regulation of day activity and night sleep. Reduced IIS increases day activity through AKH, dFOXO, and Octopamine signalling, whereas IIS regulation of night sleep is dependent on TOR and S6K activity, and dopaminergic signaling through DAT activity. Blue ovals indicate pharmacological treatments used in this study. Blue arrows indicate activation, red blocked arrows indicate inhibition, and dashed lines indicate putative interactions.
(TIF)

## Abbreviations

3IY: 3-Iodo-L-tyrosine
4E-BP: eukaryotic translation initiation factor 4E-binding protein
AKH: adipokinetic hormone
AkhR: adipokinetic hormone receptor
AKT: protein kinase B
atg: autophagy related
cAMP: cyclic adenosine monophosphate
*C. elegans*: *Caenorhabditis elegans*
CHX: cycloheximide
CNS: central nervous system
da-Gal4: daughterless-Gal4
*daf-16*: *dauer defective-16*
DAT: dopamine transporter
DD: dark∶dark schedule
dilp: *Drosophila* insulin-like peptide
DopR: dopamine receptor
FOXO: forkhead Box O
GLM: generalized linear model
IGF: insulin-like growth factor
IIS: Insulin/IGF-like signalling
INR^DN^: insulin receptor dominant negative
LD: light∶dark schedule
METH: methamphetamine
mRNA: messenger RNA
QRT-PCR: quantitative real-time polymerase chain reaction
RNA Pol II: RNA polymerase II
Rpl32: ribosomal protein L32
S6K: S6 kinase
SYA: sugar/yeast/agar
TOR: target of rapamycin
TORC: target of rapamycin complex
UAS: upstream activating sequence
w^Dah^: white^Dahomey^
