# Supplementary figures and images for: Lowered Insulin Signalling Ameliorates Age-Related Sleep Fragmentation in Drosophila
Source: PLoS Biol. 2014 Apr 1;12(4):e1001824. doi: 10.1371/journal.pbio.1001824 (PMC3972082; doi:10.1371/journal.pbio.1001824)

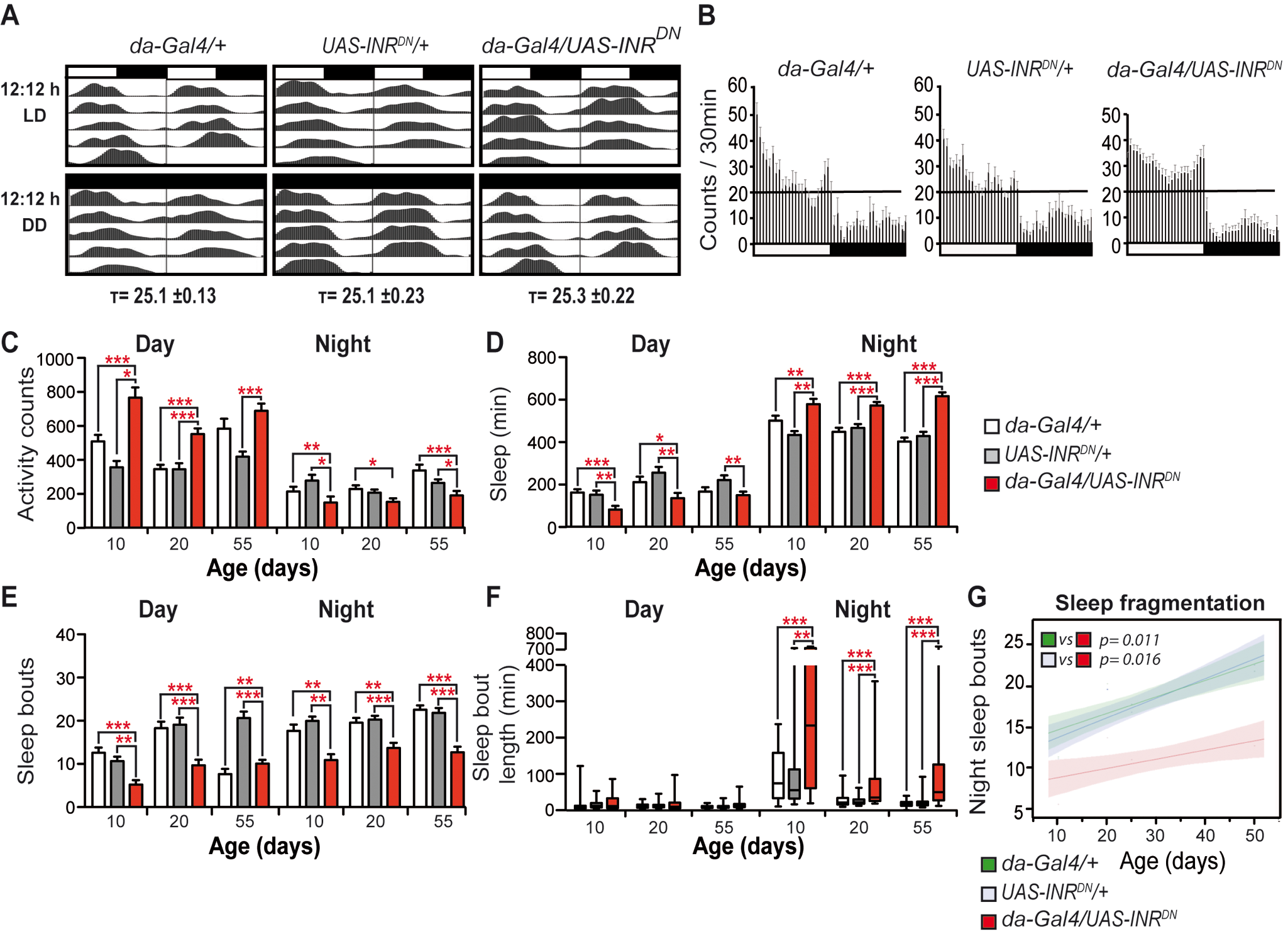

Supplement: Figure S1 — Altered activity and sleep patterns in IIS mutants. (A) Activity over 10 d (2 d per horizontal line) of 10-d-old da-Gal4/UAS-INRDN flies and controls under 12∶12 h LD (indicated by white and black bars) and 12∶12 h DD (n = 11 for all genotypes). Mean free run period (τ) in DD ± s.e.m. (B) Average activity count data (30 min bins) over a 24-h cycle under 12∶12 h LD (indicated by white and black bars) for da-Gal4/UAS-INRDN flies and controls (n = 23 for all genotypes). (C) da-Gal4/UAS-INRDN flies were more active by day and less active at night, (D) slept less or the same during the day and more during the night, (E) had fewer night sleep bouts, and (F) significantly increased night sleep bout duration, compared to control flies. (G) Control flies, but not da-Gal4/UAS-INRDN, showed a significant age-related increase in night sleep bouts (ages 10 d, 20 d, 25 d, and 55 d). (C–G) n = da-Gal4/+, n = 28, 32, 24, and 29 for ages 10 d, 20 d, 25 d, and 55 d, respectively; UAS-INRDN/+ n = 25, 31, 23, and 23 for ages 10 d, 20 d, 25 d, and 55 d, respectively; and da-Gal4/UAS-INRDN n = 27, 30, 31, and 49 for ages 10 d, 20 d, 25 d, and 55 d, respectively. Kruskal Wallis test with Dunn's multiple comparison test (selected pairs). ***p<0.001, **p<0.01, and *p<0.05. Error bars represent s.e.m. (TIF) [file pbio.1001824.s001.tif]

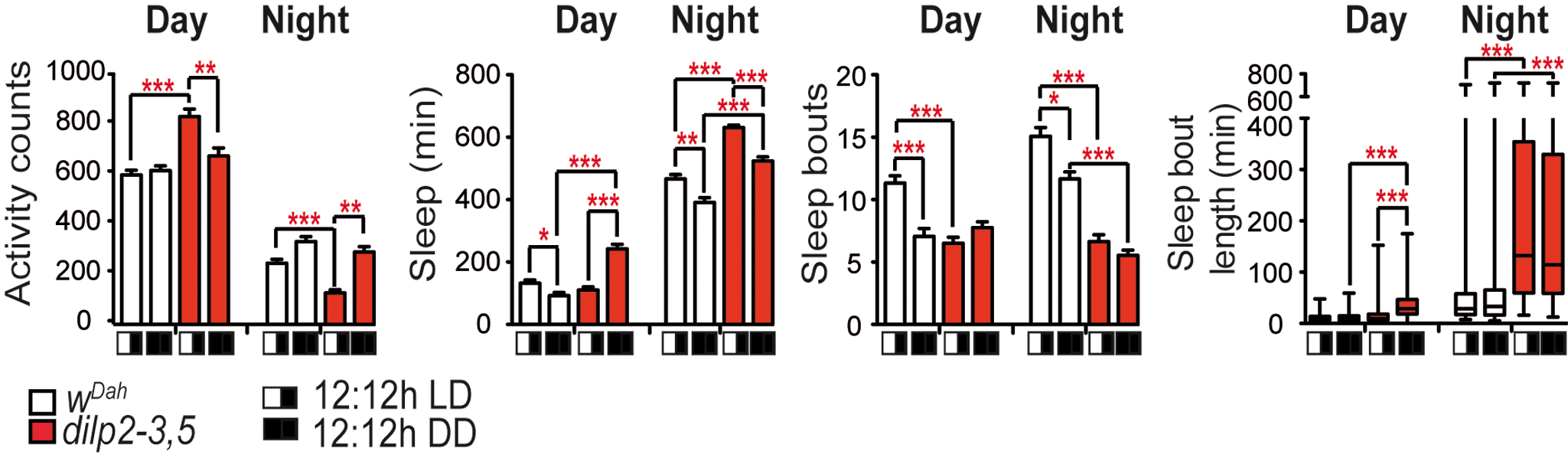

Supplement: Figure S2 — Effect of light on activity and sleep of dilp2-3,5 mutants. Activity and sleep of dilp2-3,5 mutants and controls under 12∶12 hLD and 12∶12 h dark∶dark (DD) conditions. (A) Increased day activity of dilp2-3,5 mutants was dependent on light and was lost under DD conditions. (B) In contrast to controls, which had reduced day sleep in DD conditions, dilp2-3,5 mutants had increased day sleep in DD. (C) Sleep bout number was not affected by DD in dilp2-3,5 mutants, but was decreased in controls. (D) dilp2-3,5 mutants, but not controls, had increased day sleep bout length in DD. GLM showed that dilp2-3,5 mutants had a significantly different response to light conditions in day activity (p = 0.0007), day sleep (p = <0.0001), day bout number (p = 0.0002), and day bout length (p = <0.0001) compared to controls. In contrast, no significant differences were seen in night behaviours (activity p = 0.1014, sleep p = 0.682, bout number p = 0.2019, bout length p = 0.7425). Data represent two independent experiments that were pooled: wDah n = 126, dilp2-3,5 n = 120. Ten-day-old flies, LD; 13-d-old, DD. Kruskal Wallis test with Dunn's multiple comparison test (selected pairs). ***p<0.001, **p<0.01, and *p<0.05. Error bars represent s.e.m. (TIF) [file pbio.1001824.s002.tif]

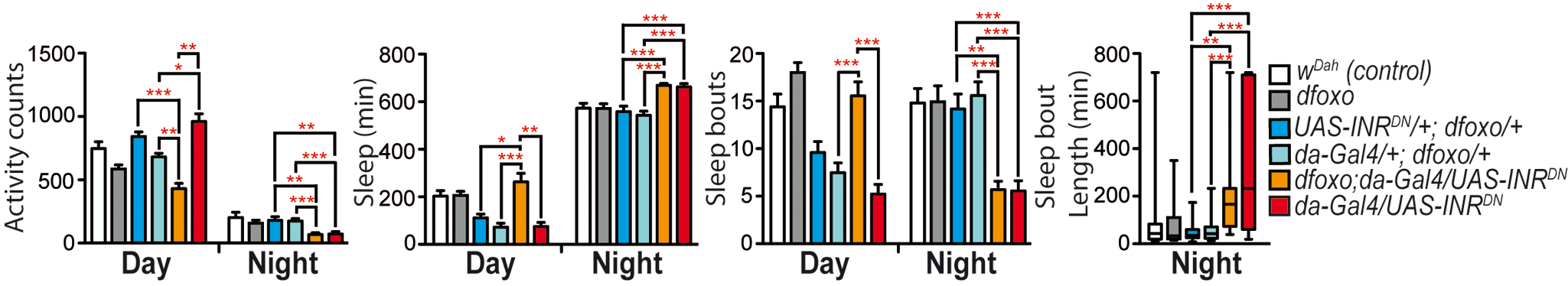

Supplement: Figure S3 — Loss of dfoxo affected daytime activity and sleep phenotypes of INRDN flies. Independent experiment verifying activity and sleep phenotypes in Figure 2 (age 10 d). dfoxo indicates the dfoxoΔ94 allele (wDah n = 30, dfoxo n = 27, UAS-INRDN/+;dfoxo/+ n = 22, da-Gal4/+;dfoxo/+ n = 32, da-Gal4/UAS-INRDN;dfoxo n = 24, da-Gal4 n = 27). Kruskal Wallis test with Dunn's multiple comparison test (selected pairs). ***p<0.001, **p<0.01, and *p<0.05. Error bars represent s.e.m. (TIF) [file pbio.1001824.s003.tif]

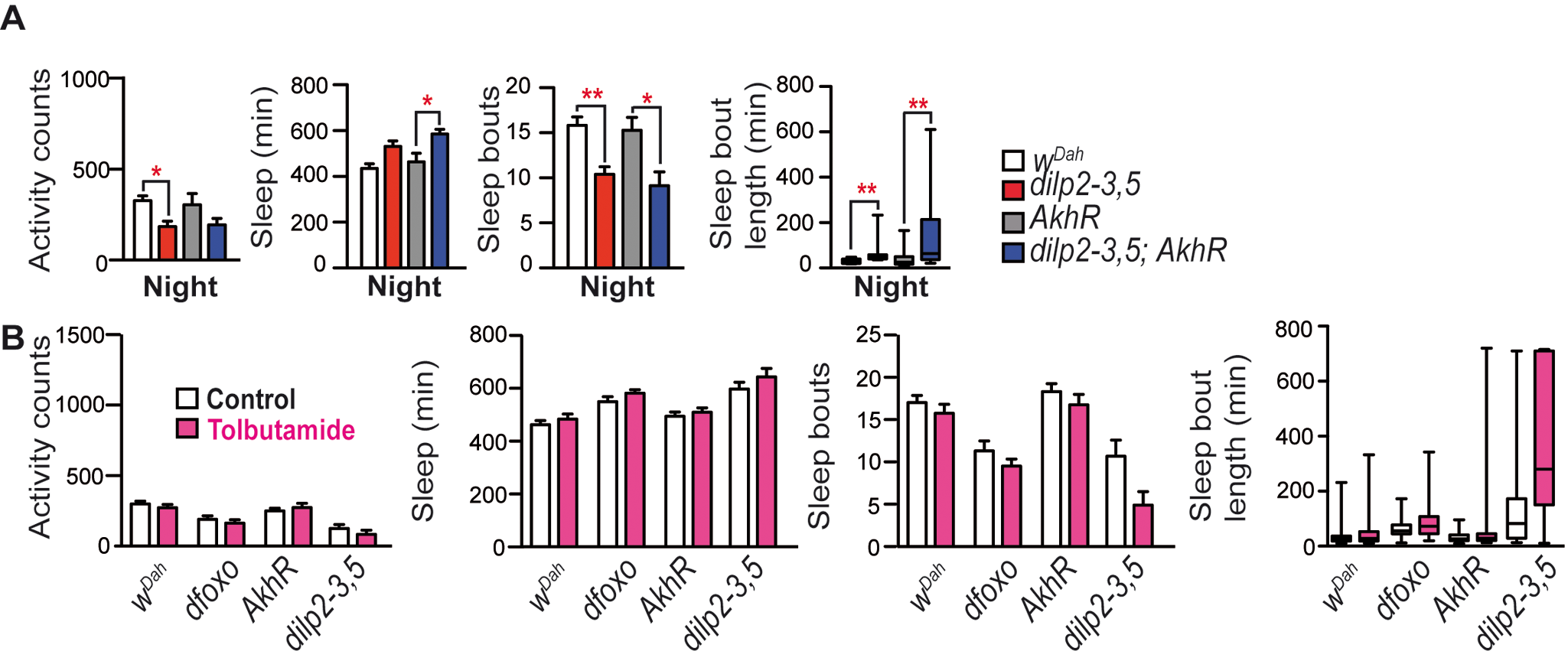

Supplement: Figure S4 — Day hyperactivity, not nighttime behaviours, of IIS mutants is mediated through AkhR. (A) Nighttime behaviour data of Figure 3A. (B) Nighttime behaviour data of Tolbutamide treated flies in Figure 3B. Kruskal Wallis test with Dunn's multiple comparison test (selected pairs). ***p<0.001, **p<0.01, and *p<0.05. Error bars represent s.e.m. (TIF) [file pbio.1001824.s004.tif]

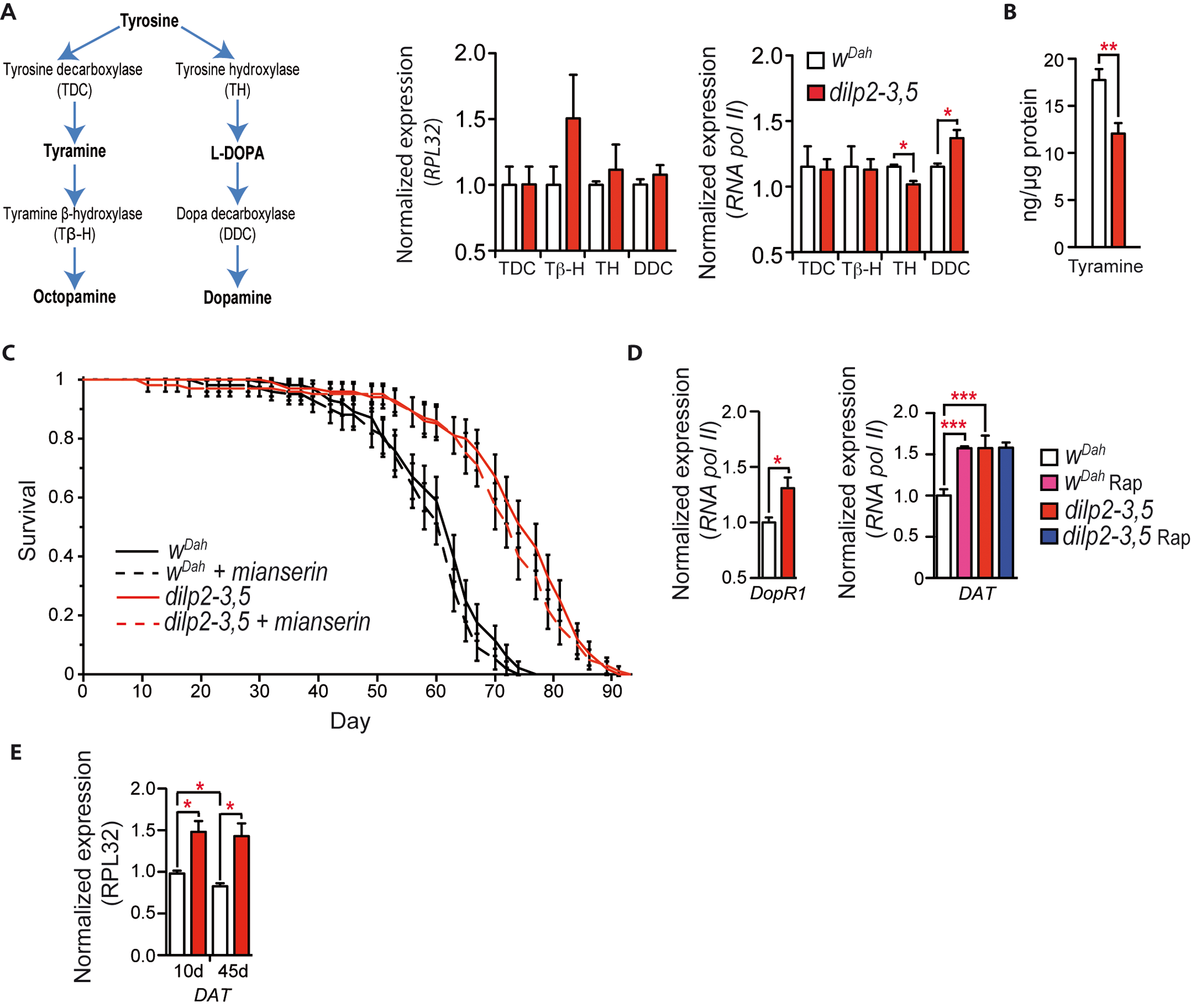

Supplement: Figure S5 — Bioamine biosynthetic enzyme expression level is independent of IIS. (A) Bioamine biosynthetic pathways and QRT-PCR analysis of biosynthetic enzyme expression in dilp2-3,5 mutant heads compared to controls (age 10 d), normalized to Rpl32 (n = 6) or RNA pol II (n = 3) expression. (B) Mass spectrometry measurement of tyramine levels in head extracts (age 35 d, wDah n = 7, dilp2-3,5 n = 8). (C) Survival analysis of wDah and dilp2-3,5 treated with mianserin (0.2 mg/ml) or control food. Significance determined by Log-rank test (n = 100 for all genotypes/treatments). (D) QRT-PCR analysis normalized to RNApolII expression matching dataset in Figure 6C and E. (E) QRT-PCR analysis of DAT expression in aged dilp2-3,5 mutant heads compared to controls, normalized to 10-d-old controls and Rpl32 (n = 3) expression. (TIF) [file pbio.1001824.s005.tif]

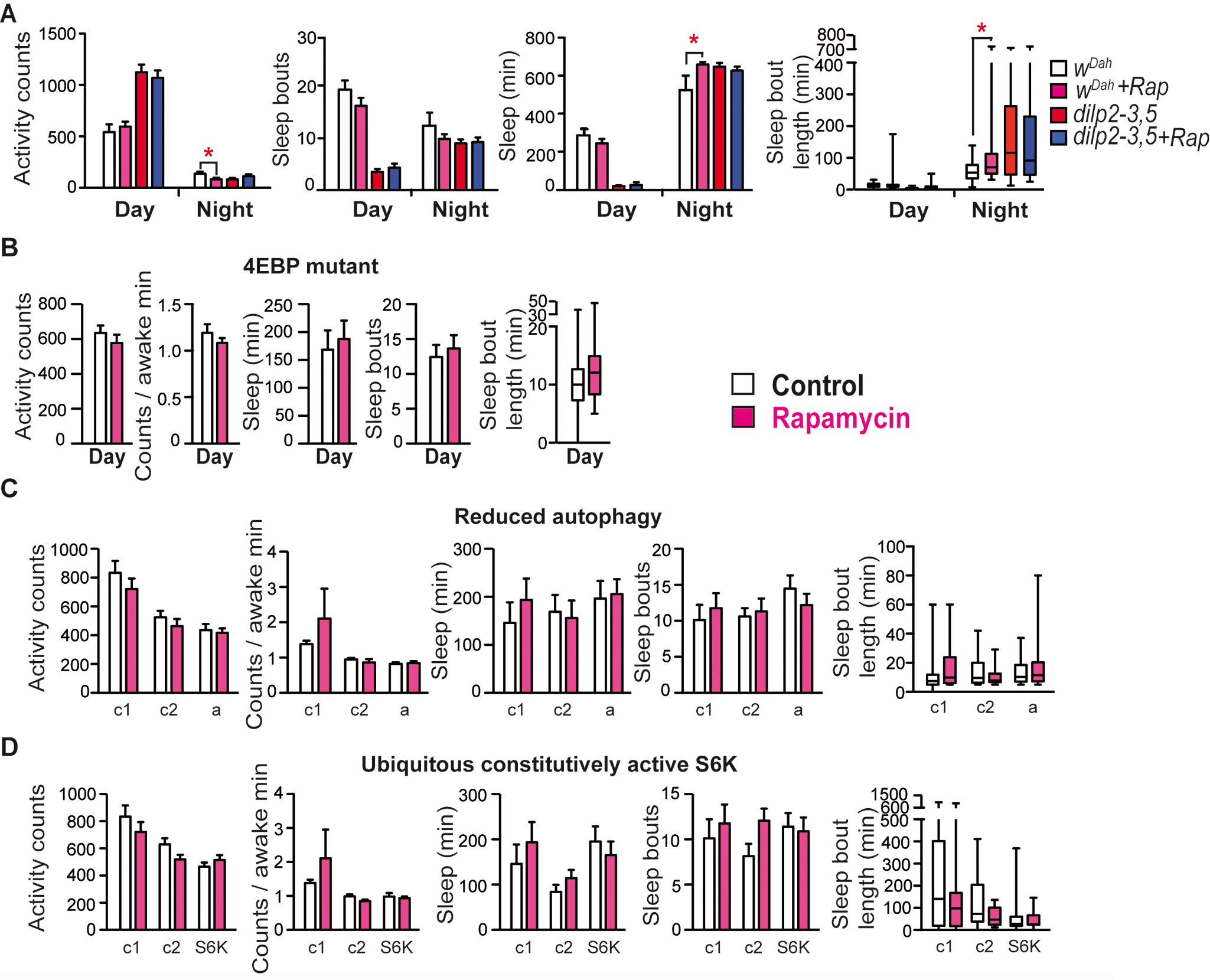

Supplement: Figure S6 — Effect of rapamycin on daytime behaviour of IIS/TOR signalling components. (A) Chronic rapamycin treatment (9 d) does not affect activity and sleep of IIS mutants (age 10 d, n = wDah control/rapamycin 21/23, dilp2-3,5 control/rapamycin 22/21), (B) 4EBP mutants (n = 19/19), (C) flies with reduced autophagy (da-Gal4/UAS-ATG5-RNAi (a) (n = 20/17)) or genetic controls (da-Gal4/+ (c1) (n = 20/21) and UAS-ATG5-RNAi/+ (c2) n = 23/19), (D) flies ubiquitously expressing constitutively active S6K (da-Gal4/UAS-S6KSTDETE (S6K) n = 20/17) or genetic controls (da-Gal4/+ (c1), n = 20/21, and UAS-S6KSTDETE/+ (c2), n = 20/18). Kruskal Wallis test with Dunn's multiple comparison test (selected pairs). ***p<0.001, **p<0.01, and *p<0.05. Error bars represent s.e.m. (Daytime behaviours from Figure 5D–F). (TIF) [file pbio.1001824.s006.tif]

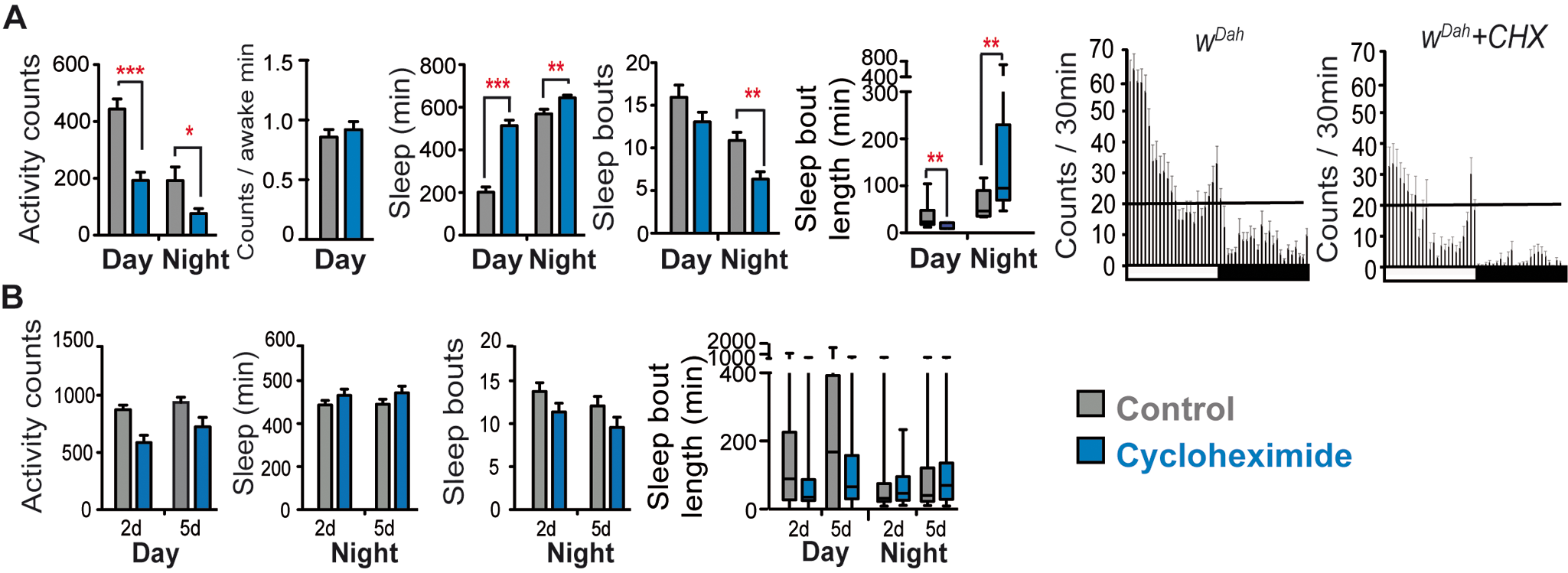

Supplement: Figure S7 — Cycloheximide affected both day and night behaviour. (A) Cycloheximide (CHX) treatment (17 mM) reduced day activity and increased day sleep but not wakefulness or day sleep bouts (age 10 d, n = 15/15 control/CHX treated). (B) Extended CHX treatment (5 d) did not alter behavior beyond that of 2 d treatment (n = control 2/5-d-old 42/43, CHX treated 2/5-d-old 29/28). Kruskal Wallis test with Dunn's multiple comparison test (selected pairs). ***p<0.001, **p<0.01, and *p<0.05. Error bars represent s.e.m. (TIF) [file pbio.1001824.s007.tif]

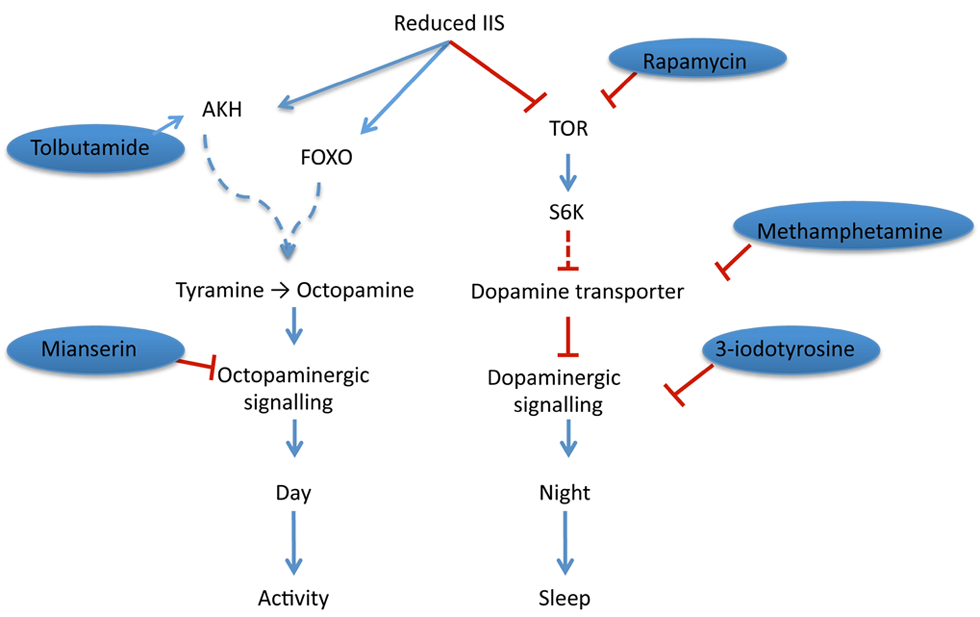

Supplement: Figure S8 — Model depicting IIS regulation of day activity and night sleep. Reduced IIS increases day activity through AKH, dFOXO, and Octopamine signalling, whereas IIS regulation of night sleep is dependent on TOR and S6K activity, and dopaminergic signaling through DAT activity. Blue ovals indicate pharmacological treatments used in this study. Blue arrows indicate activation, red blocked arrows indicate inhibition, and dashed lines indicate putative interactions. (TIF) [file pbio.1001824.s008.tif]
